# Integrated metabolome and transcriptome analysis of *Magnolia champaca* identifies biosynthetic pathways for floral volatile organic compounds

**DOI:** 10.1186/s12864-017-3846-8

**Published:** 2017-06-14

**Authors:** Savitha Dhandapani, Jingjing Jin, Vishweshwaran Sridhar, Rajani Sarojam, Nam-Hai Chua, In-Cheol Jang

**Affiliations:** 1 Temasek Life Sciences Laboratory, 1 Research Link, National University of Singapore, Singapore, 117604 Singapore; 2 Department of Biological Sciences, National University of Singapore, Singapore, 117543 Singapore; 3 Laboratory of Plant Molecular Biology, The Rockefeller University, 1230 York Avenue, New York, NY 10065 USA

**Keywords:** *Magnolia champaca*, Metabolome, Transcriptome, Floral volatile organic, Compounds, Biosynthetic pathways, Terpene synthase, Volatile esters, (R)-linalool

## Abstract

**Background:**

*Magnolia champaca*, commonly known as champak is a well-known tree due to its highly fragrant flowers. Champak floral scent is attributed to a complex mix of volatile organic compounds (VOCs). These aromatic flowers are widely used in flavors and fragrances industry. Despite its commercial importance, the VOC biosynthesis pathways in these flowers are largely unknown. Here, we combine metabolite and RNA sequencing (RNA-seq) analyses of fully opened champak flowers to discover the active VOC biosynthesis pathways as well as floral scent-related genes.

**Results:**

Volatile collection by headspace method and analysis by gas chromatography-mass spectrometry (GC-MS) identified a total of 43 VOCs from fully opened champak flowers, of which 46.9% were terpenoids, 38.9% were volatile esters and 5.2% belonged to phenylpropanoids/benzenoids. Sequencing and de novo assembly of champak flower transcriptome yielded 47,688 non-redundant unigenes. Transcriptome assembly was validated using standard polymerase chain reaction (PCR) based approach for randomly selected unigenes. The detailed profiles of VOCs led to the discovery of pathways and genes involved in floral scent biosynthesis from RNA-seq data. Analysis of expression levels of many floral-scent biosynthesis-related unigenes in flowers and leaves showed that most of them were expressed higher in flowers than in leaf tissues. Moreover, our metabolite-guided transcriptomics, in vitro and in vivo enzyme assays and transgenic studies identified (R)-linalool synthase that is essential for the production of major VOCs of champak flowers, (R)-linalool and linalool oxides.

**Conclusion:**

As our study is the first report on transcriptome analysis of *Magnolia champaca*, this transcriptome dataset that serves as an important public information for functional genomics will not only facilitate better understanding of ecological functions of champak floral VOCs, but also provide biotechnological targets for sustainable production of champak floral scent.

**Electronic supplementary material:**

The online version of this article (doi:10.1186/s12864-017-3846-8) contains supplementary material, which is available to authorized users.

## Background

Flowers emit a large group of volatile organic compounds (VOCs) that play crucial roles in interactions with other organisms. In general, floral VOCs are considered not only to serve to attract pollinators to ensure successful reproduction, but also to act as defense agents against microbes and herbivores [1]. Most of floral VOCs that are synthesized on the petals of the flowers fall into three classes of VOCs namely terpenoids, phenylpropanoids/benzenoids and volatile esters [2]. The quality and quantity of floral VOCs emitted determine floral scents and are also vital in determining the economic value of flowering plants as well as its usage in the flavors and fragrances industry.

Terpenoids, the largest class of VOCs, are produced and emitted by a number of floral species such as snapdragon and ylang ylang [3, 4]. In plants, biosynthesis of terpenoids involves three steps: (1) production of C_5_ isoprenoid precursors isopentenyl diphosphate (IPP) and dimethylallyl diphosphate (DMAPP) by two compartmentally separated pathways: the methylerythritol 4-phosphate (MEP) pathway in plastids and the mevalonate (MVA) pathway in the cytosol, (2) condensation of IPP and DMAPP into geranyl diphosphate (GPP, C_10_), farnesyl diphosphate (FPP, C_15_), and geranylgeranyl diphosphate (GGPP, C_20_), the precursors for the production of mono-, sesqui- and diterpenes, respectively and (3) generation of diverse terpene structures by the final cyclization and oxidation steps carried out by the terpene synthases (TPS) and cytochrome P450s (CYP450) [5]. A large number of TPSs involved in floral scent production have been characterized in a variety of plants [1].

Volatile esters are also known to contribute to the aroma of many fruits and flowers. For instance, volatile esters such as methyl hexanoate, ethyl hexanoate, methyl jasmonate and methyl tiglate contribute profoundly to the total floral scent composition of many plant species [2] whereas hexyl acetate, ethyl 2-methylbutanoate and methyl 3-methylvalerate have been reported to be key flavor constituents of many fruits including apple [6] and strawberry [7]. They can be derived from fatty acids or from branched-chain and aromatic amino acids [8, 9]. Conversion of fatty acids to volatile esters occur via three processes: α-oxidation, β-oxidation and lipoxygenase (LOX) pathway [10]. On the other hand, the first step in the conversion of amino acids to volatile esters is a deamination step catalyzed by aminotransferases resulting in α-ketoacids. α-ketoacids can be converted into (1) carboxylic acids via oxidative decarboxylation, (2) aldehydes via decarboxylation and (3) α-hydroxyacids via reduction by a multi-subunit complex of enzymes named as α-ketoacid dehydrogenase complex [11]. The last step in the formation of volatile esters is the esterification of the aldehydes, acids and alcohols formed by the above-mentioned processes by a class of enzymes called alcohol acyl transferases (AAT) [12].

The initial substrate for the biosynthesis of phenylpropanoids and benzenoids is phenylalanine, which is provided by the shikimate pathway [13]. Production and emission of phenylpropanoids/benzenoids are highly regulated both temporally and spatially [14]. Many phenylpropanoids/benzenoids biosynthetic enzymes have been identified and functionally characterized [14, 15]. However, the regulatory mechanisms involved in their production are relatively unknown as compared to terpenoids biosynthesis.

*Magnolia champaca*, commonly known as champak is a tall evergreen aromatic tree of the Magnoliaceae family. Since various tissues of champak have been reported to possess a broad range of medicinal properties, they are traditionally used in the treatment of various diseases such as cephalalgia, ophthalmia, gout, rheumatism and microbial infections [16]. In addition, the tree has commercial value for its strongly diffusive fragrant flowers which are widely used in cosmetic industries for the production of perfumes and essential oils due to the high amounts of VOCs [17]. The composition of champak essential oil has been investigated previously showing that it varied widely due to various factors including extraction method and flower maturity [18–20]. Although the reported percentages of the compounds were different, champak flowers mainly produced linalool and linalool oxides, 2-phenyl ethanol, methyl anthranilate, indole and methyl linoleate [18–20].

Despite the overall popularity of the champak, to our knowledge very little DNA sequences are available on GenBank for this species. The availability of DNA sequences may provide an opportunity to identify genes involved in biosynthesis of floral VOCs. Identifying and investigating the regulatory mechanisms behind floral VOCs biosynthesis is essential to understand their roles in plants and to improve VOC production through metabolic engineering. Here, we present the chemical composition and the transcriptome data from fully opened champak flowers, which were comprehensive enough to discover and analyze major secondary metabolite pathways associated with floral scent production. In addition, we identified the genes encoding the first and the last enzymes of the MEP pathway, DXS and HDR along with the (R)-linalool synthase, which makes substantial contribution to the fragrance of champak essential oil.

## Results

### Chemical composition profile of champak flowers

**Fig. 1:**
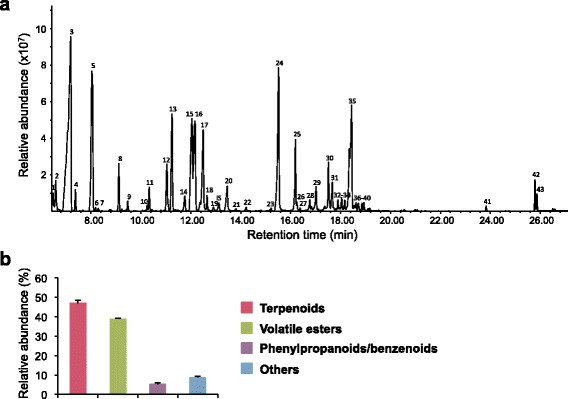
VOCs emitted from champak flowers. **a** Gas chromatogram of champak floral VOCs. The peak numbers counted in the gas chromatogram are identical to the compounds listed in Table 1. IS, internal standard, camphor. **b** Relative abundance of different classes of champak floral VOCs. Data used in the percentage calculation were obtained from the average of three replicates. Error bars indicate standard deviation (SD; *n* = 3)

A previous report on the floral VOCs of *Michelia alba*, one of the species in *Magnoliaceae* family, showed the characteristic *M. alba* fragrance to be intense from half bloom flowers to fully opened flowers [19]. To investigate the VOCs profile of fully opened champak flowers grown in Singapore, we analyzed the headspace volatiles emitted from champak flowers by GC-MS. It was observed that the VOCs emitted from champak flowers consisted of approximately 43 chemical compounds, which can contribute towards the floral scent (Fig. 1a and Table 1). These diverse VOCs emitted from champak flowers can be broadly classified into three classes: terpenoids (46.9%), volatile esters (38.9%) and phenylpropanoids/benzenoids (5.2%) (Fig. 1b). Out of 46.9% of terpenoids identified, 25.8% were monoterpenes and their derivatives, whereas 21.1% were sesquiterpenes. Five monoterpenoids, β-myrcene, β-ocimene, β-linalool, neo-allo-ocimene and α-terpineol were found in champak VOCs profile. Of these, β-linalool and its oxides such as *trans*-furanoid linalool oxide, *cis*-furanoid linalool oxide and *trans*-pyranoid linalool oxide were the most abundant components comprising about 17.5% of the total volatile composition and were followed by *cis*- and *trans*-forms of β-ocimene (6.8%). These dominant monoterpenes may contribute significantly to floral scent of champak. Although we found 15 sesquiterpenes accounting for ~21% of total volatiles, many of them were emitted in minor quantities (< 1%) except β-cubebene (8.6%), δ-elemene (3.2%), isogermacrene-D (2.3%), β-copaene (1.5%) and β-bourbonene (1.4%).

**Table 1:** Volatile organic compounds composition emitted from champak flowers

No.^a^	Compound	RT (min)^b^	RI^c^	Formula	ng.g^−1^ flower.h^-1 d^	RA (%)
1	Ethyl propanoate	6.492	703	C_5_H_10_O_2_	1.96 ± 0.13	0.56
2	2-Methylbutanoate	6.593	713	C_5_H_10_O_2_	4.14 ± 0.40	1.19
3	Methyl 2-methylbutanoate	7.188	744	C_6_H_12_O_2_	80.69 ± 4.34	23.16
4	Ethyl butanoate	7.383	792	C_6_H_12_O_2_	1.93 ± 0.40	0.55
5	Ethyl 2-methylbutanoate	8.048	841	C_7_H_14_O_2_	32.66 ± 3.64	9.38
6	3-Methylbutanal oxime	8.189	851	C_5_H_11_NO	0.57 ± 0.01	0.16
7	Methyl tiglate	8.297	859	C_6_H_10_O_2_	0.43 ± 0.01	0.12
8	Methyl hexanoate	9.122	906	C_7_H_14_O_2_	5.17 ± 0.57	1.48
9	Propyl 2-methylbutanoate	9.477	937	C_8_H_16_O_2_	1.44 ± 0.02	0.41
10	β-Myrcene	10.261	982	C_10_H_16_	0.84 ± 0.01	0.24
11	Ethyl hexanoate	10.338	989	C_8_H_16_O_2_	2.16 ± 0.53	0.62
12	(Z)-β-Ocimene	11.045	1031	C_10_H_16_	7.71 ± 0.78	2.21
13	(E)-β-Ocimene	11.25	1043	C_10_H_16_	16.10 ± 1.42	4.62
14	(Z)-Furanoid linalool oxide	11.763	1063	C_10_H_18_O_2_	2.91 ± 0.1	0.84
15	(E)-Furanoid linalool oxide	12.047	1079	C_10_H_18_O_2_	27.07 ± 0.92	7.77
16	β-Linalool	12.168	1092	C_10_H_18_O	24.71 ± 1.54	7.09
17	Phenylethyl alcohol	12.497	1103	C_8_H_10_O	17.12 ± 1.83	4.91
18	Neo-allo-ocimene	12.656	1125	C_10_H_16_	1.91 ± 0.26	0.55
19	Benzyl nitrile	12.912	1140	C_8_H_7_N	0.68 ± 0.14	0.19
20	(E)-Pyranoid linalool oxide	13.461	1173	C_10_H_18_O_2_	6.34 ± 0.29	1.82
21	α-Terpineol	13.809	1185	C_10_H_18_O	0.61 ± 0.04	0.17
22	Geranyl methyl ether	14.225	1219	C_11_H_20_O	0.99 ± 0.03	0.28
23	Geranyl ethyl ether 1	15.225	1264	C_12_H_22_O	0.73 ± 0.02	0.21
24	Indole	15.519	1285	C_8_H_7_N	30.03 ± 2.81	8.62
25	δ-Elemene	16.194	1342	C_15_H_24_	11.13 ± 0.71	3.19
26	Methyl anthranilate	16.25	1346	C_8_H_9_NO_2_	0.54 ± 0.01	0.15
27	α-Copaene	16.382	1355	C_15_H_24_	0.41 ± 0.01	0.12
28	α-Ylangene	16.771	1380	C_15_H_24_	2.50 ± 0.23	0.72
29	β-Bourbonene	17.021	1396	C_15_H_24_	4.89 ± 0.30	1.40
30	Isogermacrene-D	17.518	1430	C_15_H_24_	7.99 ± 0.32	2.29
31	β-Copaene	17.67	1441	C_15_H_24_	5.38 ± 0.26	1.55
32	γ-Amorphene	17.898	1456	C_15_H_24_	1.72 ± 0.14	0.49
33	γ-Muurolene	18.048	1466	C_15_H_24_	2.28 ± 0.18	0.65
34	(Z)-Muurola-4(14),5-diene	18.172	1475	C_15_H_24_	1.73 ± 0.07	0.50
35	β-Cubebene	18.443	1478	C_15_H_24_	29.87 ± 3.42	8.57
36	δ-Guaiene	18.542	1500	C_15_H_24_	0.84 ± 0.05	0.24
37	α-Muurolene	18.622	1506	C_15_H_24_	1.48 ± 0.04	0.43
38	δ-Cadinene	18.717	1513	C_15_H_24_	1.3 ± 0.06	0.37
39	γ-Cadinene	18.862	1523	C_15_H_24_	0.99 ± 0.03	0.28
40	β-Cadinene	18.939	1529	C_15_H_24_	1.22 ± 0.02	0.35
41	Methyl palmitate	23.836	1908	C_17_H_34_O_2_	0.58 ± 0.06	0.17
42	Methyl linoleate	25.792	2080	C_19_H_34_O_2_	2.83 ± 0.30	0.81
43	Methyl linolenate	25.872	2087	C_19_H_32_O_2_	1.83 ± 0.17	0.52

^a^Compounds are listed in the same order as their elution from HP-5MS column

^b^Retention Time in minutes

^c^Retention Indices calculated using C_7_-C_30_ n-alkanes on HP-5MS column

^d^Calculated from three independent readings

The pleasant fragrance of flowers such as *Wisteria*, orchids and lilies is often due to the presence of a particular volatile ester or a mixture of several volatile esters [2]. We found volatile esters as the second major class of champak floral volatiles, which constituted 38.9% of total VOCs (Fig. 1 and Table 1). Particularly, different derivatives of butanoic acid contributed significantly (35.9%) to the total VOCs of champak flowers. Of these, methyl 2-methylbutanoate solely constituted 23.2% of total floral volatiles. In addition, ester forms of hexanoic acid, propanoic acid, linoleic acid and linolenic acid were also among the champak floral VOCs. Phenylethyl alcohol (4.9%) was the only phenylpropanoid identified and minor amounts of two benzenoids, benzyl nitrile and methyl anthranilate were also part of the champak floral volatile composition.

VOCs composition of fully opened champak flowers was analyzed at several time points of the year. However, we could not see significant variation in volatile profile of these flowers, as the weather conditions in Singapore do not fluctuate considerably throughout the year due to the geographical location of Singapore.

### RNA-seq, de novo assembly, and annotation of transcriptome

To identify genes involved in the biosynthesis of floral VOCs from champak flowers, we sequenced RNA libraries derived from flowers using an Illumina HiSeq2000. Illumina sequencing from RNA-seq libraries yielded approximately 169.6 million reads of 101 bp length. After eliminating the adaptor sequences, 122.8 million reads with 47% GC content were obtained. FastQC [21] analysis showed that 73.2% of the total sequences were of quality above Q30 (Additional file 1: Figure S1a). As the genomic sequence of champak is unavailable, the reads were assembled de novo into a total of 47,688 unigenes with N50 of 1814 bp using Trinity method ([22]; Additional file 2; Table 2). Total number of assembled unigenes might be overestimated due to the absence of reference genome. It should be noted that more than 5000 unigenes were observed in the length range of 200–300 bp, most of which were not annotated by public databases. A large number of unannotated unigenes may indicate (1) novel genes specifically expressed in champak flowers, (2) incomplete sequencing of some very low expressed genes yielding two/more unigenes, (3) presence of non-coding RNAs [23, 24].

**Table 2:** Overview of RNA-seq de novo assembly results

No. Reads	No. Unigenes	N50 (bp)	No. Annotated	% Annotation
169,602,594	47,688	1814	27,043	56.7

**Fig. 2:**
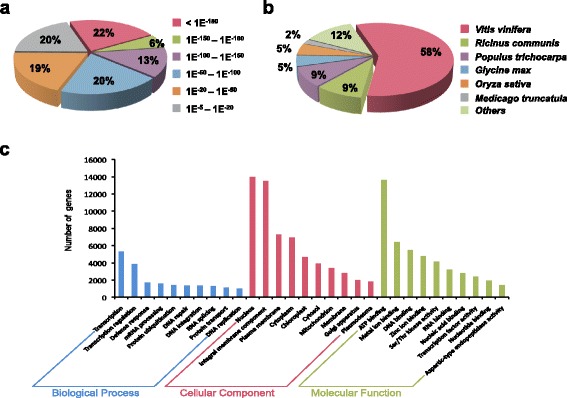
Analysis of champak unigenes. **a** E-value distribution of top BLASTX hits. **b** Species distribution based on the top BLASTX hits. **c** Top 10 GO terms from all three categories

Of the 47,688 unigenes, 27,043 (56.7%) were annotated using BLASTX (E-value cut-off of 10^−5^) against four protein databases: National Centre for Biotechnology Information (NCBI) non-redundant (nr), *Arabidopsis* (*Arabidopsis thaliana*), rice (*Oryza sativa*) and grape (*Vitis vinifera*). BLASTX analysis showed that 41% of unigenes had extremely high homology (E-value <10^−100^), 20% had high homology (10^−100^ < E-value <10^−50^) and 39% had moderate homology (10^−50^ < E-value <10^−5^) to nr database (Fig. 2a). The species distribution of the best-match result showed that the top hits for 58% of unigenes were from *Vitis vinifera*, followed by *Ricinus communis* (9%), *Populus trichocarpa* (9%), *Glycine max* (5%), *Oryza sativa* (5%) and *Arabidopsis* (1.1%) (Fig. 2b).

### Functional classification of genes

Champak unigenes were functionally classified into different Gene Ontology (GO) terms using Trinotate [25]. Classification showed that 38% of the annotated genes were involved in biological process, 29.5% in cellular component and 32.5% in molecular function (Additional file 2). We further looked at the top ten GO-terms in each of the three GO categories (Fig. 2c). Within biological process, transcription (19.6%) and regulation of transcription (14.2%) were the two dominating GO terms and they were followed by defense response, which suggests that champak flowers are probably an active tissue for secondary metabolism. Among the category cellular component, 51.6%, 50%, 26.9%, 25.6% of the annotated genes were classified into the GO-terms nucleus, integral component of membrane, plasma membrane and cytoplasm respectively. In the group of molecular function, ATP binding, metal ion binding, DNA binding and zinc binding were the principal GO-terms of molecular function comprising of 50.5%, 23.7%, 20.2% and 17.8% annotated genes respectively.

### Analysis of highly expressed genes in champak flowers

**Fig. 3:**
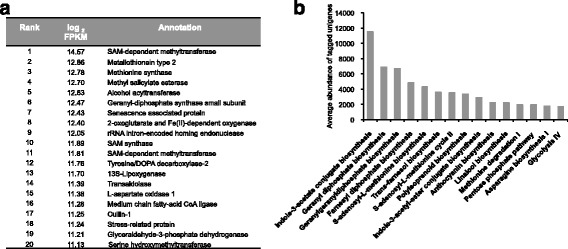
Expression and pathway analysis of champak unigenes. **a** Top 20 genes most highly expressed in champak flowers. **b** Top 15 mapped pathways annotated by Plant metabolic pathway database (PlantCyc, [30])

We investigated the top 20 transcripts that were highly expressed in champak flowers and found many of them to be involved in the biosynthesis of floral VOCs (Fig. 3a). For instance, transcript encoding for geranyl diphosphate synthase small subunit (GPS.SSU), S-adenosyl-L-methionine-dependent methyltransferases (SAMT), methionine synthase, S-adenosylmethionine synthase, 13S–lipoxygenase, medium chain fatty-acid CoA ligase and alcohol acyl transferase were all highly expressed and are known to be involved in the biosynthesis of terpenoids, benzenoids and volatile esters. This expression pattern correlates well with the result showing VOCs profile emitted from champak flowers in Fig. 1 and Table 1. Many fatty acid-derived methyl esters (FAMEs) are believed to be synthesized from polyunsaturated fatty acids via the lipoxygenase pathway [26]. Several FAMEs such as methyl palmitate, methyl linoleate and methyl linolenate were found in champak VOCs (Fig. 1 and Table 1). The highly expressed unigene encoding 13S–lipoxygenase might be responsible for their synthesis.

The second set of abundant transcripts encoded for proteins that function in plant defense. These were the metallothionein (MT), methyl salicylate esterase, stress-related protein and serine hydroxymethyltransferase (SHMT), which play a role in detoxification of heavy metals and reactive oxygen species [27], to confer immunity to plants [28] and in controlling cell damage from stress conditions [29], respectively.

### Pathway analysis of unigenes expressed in champak flowers

The top fifteen pathways with highest average Fragments Per Kilobase of transcript per Million mapped reads (FPKM) of tagged enzymes were identified using the plant metabolic pathway database (PlantCyc, [30]; Additional file 2). Figure 3b shows that majority of the top fifteen pathways were involved in secondary metabolites biosynthesis. It was apparent that pathways involved in the production of substrates (GPP, FPP, and GGPP) essential for terpenoids biosynthesis were highly expressed. In addition, genes involved in pathways leading to the biosynthesis of S-adenosyl-L-methionine, a common substrate for methyl group transfers, and its degradation were also highly expressed in the champak flowers. These results are not surprising as champak flowers produced diverse mono- and sesqui-terpenes as well as methylated volatile esters (Fig. 3b and Table 1). Interestingly, pathways for the indole-3-acetate (IAA) and indole-3-acetyl-ester conjugate biosynthesis were among the top 20. This suggests that auxin signaling probably impedes floral VOCs biosynthesis as IAA conjugates are known to be endogenous auxin inhibitors [31].

The Kyoto Encyclopedia of Genes and Genomes (KEGG) pathway database was used to identify the biological pathways active in champak flowers [32]. When the unigenes were assigned KEGG Orthology (KO) numbers using the bi-directional best-hit method in KEGG Automatic Annotation Server (KAAS) [33], a total of 6597 unigenes were mapped onto 373 KEGG pathways (Additional file 2). Carbon metabolism (ko01200), biosynthesis of amino acids (ko01230) and protein processing in endoplasmic reticulum (ko04141) were identified as the top three KEGG pathways (Additional file 1: Figure S1b). Among the top twenty KEGG pathways, terpenoid backbone biosynthesis, pentose phosphate pathway and glycolysis were found, supporting the results obtained by the plant metabolic pathway database (Fig. 3b and Additional file 1: Figure S1b). Additionally, pathway analysis using KEGG database identified 2-oxocarboxylic acid metabolism (ko01210) as one of the top pathways. This pathway includes the conversion of L-isoleucine to (S)-3-methyl-2-oxopentanoic acid, which is the first step in the proposed biosynthetic pathway of 2-methylbutanoate, methyl 2-methylbutanoate, ethyl 2-methylbutanoate and propyl 2-methylbutanoate (Additional file 1: Figure S1b and Figure S2).

**Fig. 4:**
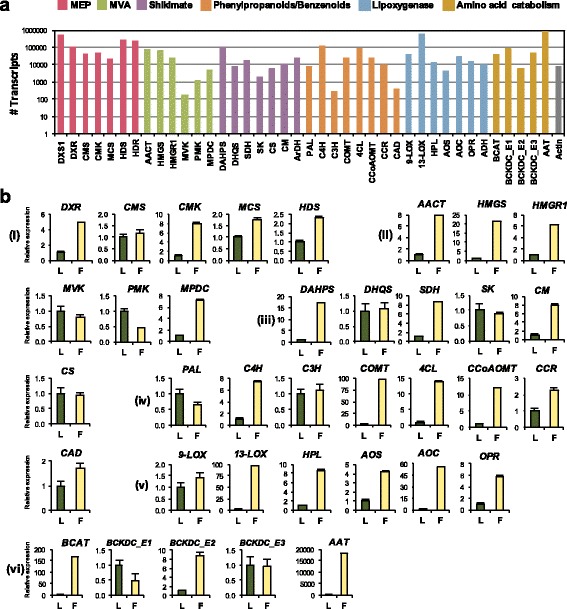
Analysis of expression levels of genes involved in floral VOCs biosynthesis. **a** Number of transcripts for individual genes involved in different pathways. Transcript counts are presented in a logarithmic scale of 10. **b** qRT-PCR analysis of representative genes from (i) MEP (ii) MVA (iii) Shikimate (iv) Phenylpropanoid/Benzenoid (v) Lipoxygenase and (vi) Amino acid catabolic pathways in leaves (L) and flowers (F). DXR, 1-deoxy-D-xylulose 5-phosphate reductoisomerase; CMS, 2-C-methyl-D-erythritol 4-phosphate cytidylyltransferase; CMK, 4-(cytidine 5′-diphospho)-2-C-methyl-D-erythritol kinase; MCS, 2-C-methyl-D-erythritol 2,4-cyclodiphosphate synthase; HDS, 4- hydroxy-3-methylbut-2-en-1-yl diphosphate synthase; AACT, acetyl-CoA acetyltransferase; HMGS, hydroxymethylglutaryl-CoA synthase; HMGR, hydroxymethylglutaryl-CoA reductase; MVK, mevalonate kinase; PMK, phosphomevalonate kinase; MPDC, mevalonate diphosphate decarboxylase; DAHPS, 3-deoxy-D-arabino-heptulosonate-7-phosphate synthase; DHQS, 3-dehydroquinate synthase; SDH, shikimate dehydrogenase; SK, shikimate kinase; CM, chorismate mutase; CS, chorismate synthase; PAL, phenylalanine ammonia lyase; C4H, cinnamate-4-hydroxylase; C3H, p-coumarate-3-hydroxylase; COMT, caffeic acid/5-hydroxyferulic acid O-methyltransferase; 4CL, 4-coumaroyl-CoA ligase; CCoAOMT, caffeoyl-CoA 3-O-methyltransferase; CCR, cinnamoyl-CoA reductase; CAD, cinnamyl alcohol dehydrogenase; 9-LOX, linoleate 9S–lipoxygenase 5; 13-LOX, linoleate 13S–lipoxygenase; HPL, hydroperoxide lyase; AOS, allene oxide synthase; AOC, allene oxide cyclase; OPR, 12-oxophytodienoate reductase; BCAT, branched-chain amino-acid aminotransferase; BCKDC_E1, pyruvate dehydrogenase E1 alpha; BCKDC_E2, dihydrolipoamide acyltransferase; BCKDC_E3, dihydrolipoamide dehydrogenase; AAT, alcohol acyl transferase

Next, we investigated the transcripts encoding enzymes for the precursor pathways related to champak floral VOCs biosynthesis. Figure 4a shows the expression profile of genes involved in MEP, MVA, shikimate, phenylpropanoids/benzenoids, lipoxygenase and amino acid catabolic pathways, which are main pathways for champak floral VOCs such as terpenoids, phenylpropanoids/benzenoids and volatile esters. Most of the genes involved in these pathways showed high abundance in the RNA-seq of champak flowers and included full length open reading frames (ORFs) with high homology to their orthologous genes from other plants (Additional file 1: Figure S3).

The expression levels of these transcripts were checked in champak flowers and leaves by quantitative real-time PCR (qRT-PCR) in order to see if they are specifically up-regulated in flowers, thus contributing to floral scent (Fig. 4b). Overall, approximately 70% of these transcripts analyzed showed at least 2-fold higher expression in flowers than leaves. Interestingly, transcripts encoding branched-chain amino acid aminotransferase (BCAT) and alcohol acyl transferase (AAT), which are involved in the biosynthesis of several volatile esters including methyl 2-methylbutanoate (Additional file 1: Figure S2) showed preferential expression in flowers than in leaves.

### Characterization of MEP pathway genes

Since monoterpenes are one of most abundant VOCs in champak flowers, we looked at genes encoding MEP pathway enzymes that are necessary to provide IPP and DMAPP for monoterpene biosynthesis in the plastids [34]. Based on the search of the champak transcriptome against orthologous sequences from other plant species, we found single copy genes of all MEP pathway enzymes except *DXS* (1-deoxy-D-xylulose 5-phosphate synthase) that encodes four isoforms in champak flower (Additional file 1: Table S1).

**Fig. 5:**
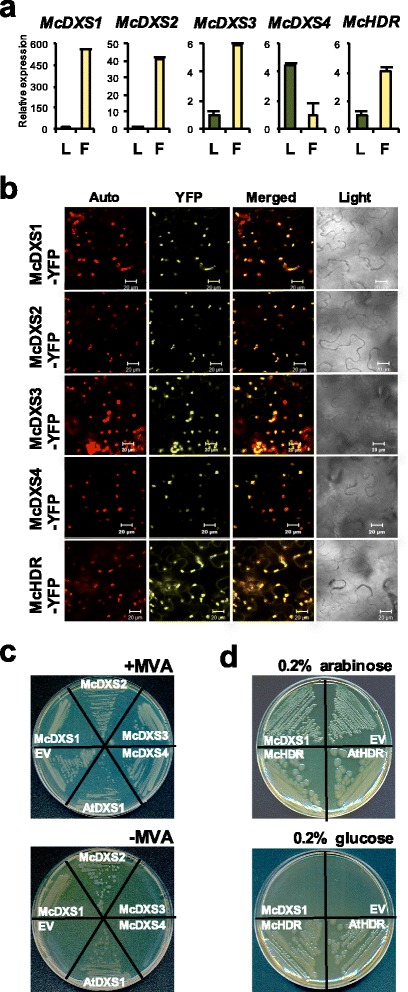
Characterization of *McDXSs* and *McHDR*. **a** Expression of champak 1-deoxy-D-xylulose 5- phosphate synthase (*McDXSs*) and 1-hydroxy-2-methyl-2-(E)-butenyl 4-diphosphate reductase (*McHDR*) genes in leaves (L) and flowers (F). **b** Subcellular localization of McDXSs and McHDR. Auto, chlorophyll autofluorescence; YFP, YFP channel image; Merged, merged image of Auto and YFP; Light, light microscopy image. Bars, 20 μm. **c** and **d** Complementation assays of champak DXS isoforms (**c**) and HDR (**d**) using *E.coli dxs* ^*−*^ and MG1655 *ara<>ispH* mutants, respectively. For DXS complementation assay, *E.coli* cells containing *Arabidopsis thaliana* DXS (AtDXS1) and empty vector (EV) were used as a positive and negative control, respectively. For the HDR complementation assay, *Arabidopsis thaliana* HDR (AtHDR) was used as a positive control while empty vector (EV) and McDXS1 were used as negative controls

We further investigated the champak *DXS* family genes and the *HDR* gene, as they are the first and last enzyme in MEP pathway, respectively. Four isoforms of DXS were found in champak flowers, which were designated *McDXS1–4* based on transcript abundance. Phylogenetic analysis based on deduced amino acid sequences of four *McDXS* cDNAs showed that McDXS1 and McDXS2 belonged to clade II and their transcripts were predominantly expressed in champak flowers than in leaves (Fig. 5a and Additional file 1: Figure S4a). Clade II DXS is involved in secondary metabolism. On the other hand, McDXS4 was the only member of clade I and McDXS3, the most divergent among the four McDXSs, was part of the most distant group - clade III. The amino acid residues essential for binding of glyceraldehyde-3-phosphate (G3P) and thiamine pyrophosphate (TPP) were conserved in McDXS1, McDXS2 and McDXS4, but not in McDXS3 (Additional file 1: Figure S4b).

Analysis of deduced amino acid sequence of *McHDR* showed that four-cysteine residues, which might be essential for the coordination of iron-sulfur bridge were well conserved ([35]; Additional file 1: Figure S5).

All four DXSs and HDR contained a transit peptide (TP) sequence at their N-terminus for plastidic targeting (Additional file 1: Table S1). In order to validate this result, we observed the subcellular localization of McDXS1–4 and McHDR. Yellow fluorescent protein (YFP) fused DXS or HDR construct was transiently expressed in *N. benthamiana* leaves using an *Agrobacterium*-mediated infiltration. Figure 5b shows that all McDXSs as well as McHDR were clearly localized in chloroplasts of *N. benthamiana* leaves.

To find out if all the four McDXSs are functionally active, we carried out complementation assay using a *dxs*-deficient *Escherichia coli* strain. The *dxs* ^*−*^ *E. coli* cells can grow normally in the presence of mevalonate but on mevalonate-free media, the cells require functional DXS for their viability [36]. Figure 5c shows that transformants harbouring McDXS1 and McDXS2 were able to complement the *dxs* ^*−*^ lines on mevalonate-free media similar to the positive control *Arabidopsis* DXS1. However, transformants containing McDXS3, MCDXS4 and empty vector as a negative control were unable to grow on mevalonate-free media even after 2d of incubation.

The enzymatic activity of McHDR was analyzed by complementation assay using a *hdr*-deficient *E. coli* strain, *MG1655 ara<>ispH* that grows normally in the presence of arabinose, but require a functional exogenous HDR to survive in the presence of glucose [37]. Figure 5d shows that *MG1655 ara<>ispH* cells transformed with *McHDR* were capable of growing well on media containing glucose, similar to the cells transformed with the positive control *Arabidopsis HDR.* But, the cells transformed with either the empty vector or *McDXS1* did not grow on media containing glucose. This result demonstrated that the *McHDR* encodes a functionally active HDR enzyme.

### Terpene synthases from champak flowers

Champak flowers emit at least twenty six different types of mono- and sesqui-terpenes and their derivatives (Fig. 1a and Table 1). We found approximately nine candidate *TPS* unigenes from the transcirptome data of champak flowers (Additional file 1: Table S2). While most candidate *TPS* transcripts were partial mRNA sequences, only one transcript contained a full-length ORF. Deduced amino acid sequence of full-length TPS showed 67% similarity with *trans*-ocimene synthase from mountain pepper (*Litsea cubeba*)*.* Interestingly, we found four partial mRNA sequences that showed high homology up to 98% to the single gene encoding β-cubebene synthase (Mg25) from *Magnolia grandiflora* [38]. Since β-cubebene was the most abundant sesqui-terpene found in champak flowers accounting for 8.57% of the total VOCs, 18.2% of terpenes, and 40.5% of sesqui-terpenes (Fig. 1a and Table 1), they were predicted to encode β-cubebene synthase.

**Fig. 6:**
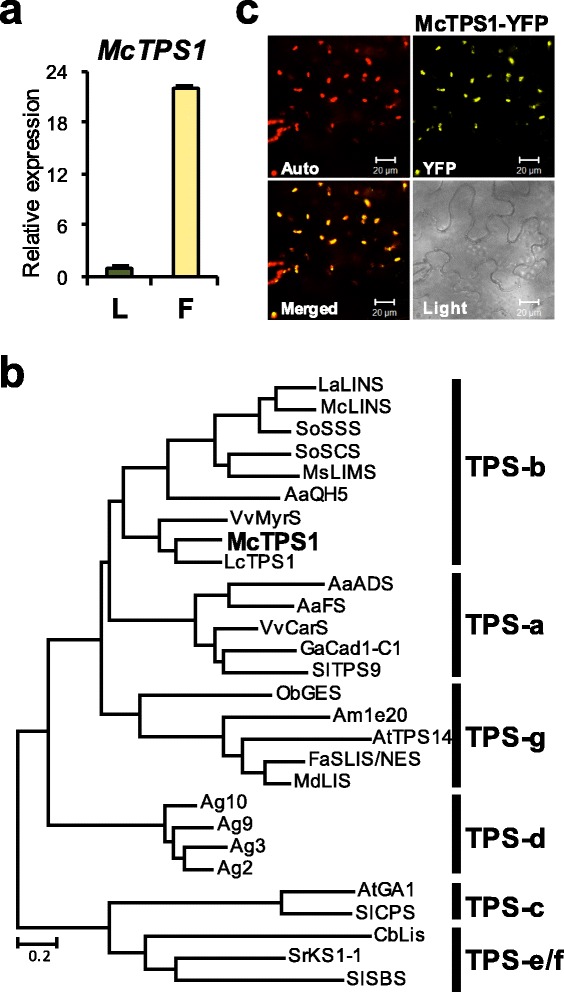
Characterization of *McTPS1*. **a** Expression of *McTPS1* in leaves (L) and flowers (F). **b** Phylogenetic analysis of McTPS1. A maximum likelihood tree was constructed using amino acid sequences of McTPS1 with other plant TPSs. Accession numbers of proteins used in phylogenetic analysis are listed in Additional file 1: Table S3. **c** Subcellular localization of McTPS1. Auto, chlorophyll autofluorescence; YFP, YFP channel image; Merged, merged image of Auto and YFP; Light, light microscopy image. Bars, 20 μm

Next, we further investigated the *TPS* gene (designated here as *McTPS1*) containing the complete ORF. *McTPS1* was predominantly expressed in flowers than in leaves (Fig. 6a). According to the phylogenetic analysis, McTPS1 was grouped into TPS-b subfamily, which commonly represents mono-TPSs (Fig. 6b; [39]). In addition to the aspartate-rich domains, DDXXD and NSE/DTE motif, which is highly conserved in plant TPSs, McTPS1 contained two distinct structural domains of the TPS-b group, a TP sequence for plastid targeting and the R(R)X_8_W motif for monoterpene cyclization at N-terminal region (Additional file 1: Figure S6). As expected, the McTPS1-YFP was localized in chloroplast of *N. benthamiana* cells (Fig. 6c), suggesting McTPS1 is a mono-TPS.

**Fig. 7:**
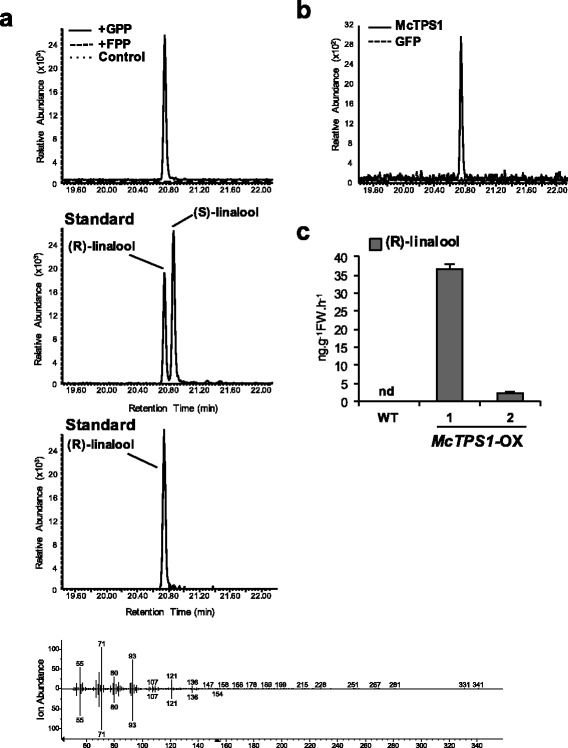
Functional characterization of McTPS1. **a** In vitro enzymatic assay of recombinant 6His-McTPS1 protein using either GPP or FPP as substrate. Control, TPS assay of the heat-inactivated 6His-McTPS1 recombinant protein with GPP. The reaction products were analyzed by chiral phase GC-MS. The peak obtained from in vitro assay was identified with the authentic standard, (±)-linalool. Mass spectra of the peak from the assay and (R)-linalool are shown at the bottom of chromatograms. **b** In vivo characterization of McTPS1 by transient expression of McTPS1 in *N. benthamiana* leaves. VOCs were collected from McTPS1 or GFP infiltrated *N. benthamiana* leaves and analyzed by chiral phase GC-MS. **c** (R)-linalool emission levels in transgenic *N. tabacum* lines overexpressing *McTPS1* (#1 and #2, McTPS1-OX) or wild-type (WT). Volatiles were collected by headspace method and analyzed by chiral phase GC-MS method. The quantity of (R)-linalool was determined by calculating the peak area of internal standard camphor (100 ng/μl). Error bars indicate SD (*n* = 2). nd, not detectable

To figure out the exact function of McTPS1, 6His-tagged McTPS1 recombinant protein was used for in vitro assays in the presence of geranyl diphosphate (GPP) and farnesyl diphosphate (FPP) as the common substrate for mono- and sesqui-TPS, respectively. Figure 7a shows that McTPS1 reacted with GPP to produce (R)-linalool, a monoterpene alcohol, whereas it did not react with FPP. As a negative control, heat denatured McTPS1 protein was added to GPP and FPP reaction mixtures and they failed to produce any terpene. Therefore, our in vitro assays identified McTPS1 as the (R)-linalool synthase, responsible for the production of the most abundant monoterpene of champak flowers. In vivo functional studies were carried out by infiltrating the leaves of *N. benthamiana* with *Agrobacterium* harboring *McTPS1*. Plants infiltrated with green fluorescent protein (GFP) served as a negative control. At 3 days post-infiltration (dpi), the volatiles were collected from the plants by push-pull headspace method and analyzed by GC-MS. *N. benthamiana* plants expressing McTPS1 emitted (R)-linalool which is consistent with those obtained in vitro whereas no linalool was detected in *N. benthamiana* plants expressing GFP (Fig. 7b). When chiral column was used to analyze the volatile composition of champak flowers, it was found that champak flowers emitted (R)-linalool almost exclusively (Additional file 1: Figure S7).

Additionally, we generated more than 10 lines of transgenic *N. tabacum* plants expressing *McTPS1* (Additional file 1: Figure S8). One-month-old T1 progenies from two independent transgenic lines expressing *McTPS1* were used for headspace collection of VOCs using a push-pull headspace collection system. Figure 7c show that (R)-linalool emission in the transgenic lines were positively correlated with expression levels of *McTPS1* while no linalool was detected in wild type (WT), which was the same result as that from transient expression in the leaves of *N. benthamiana*.

## Discussion

Our analysis on VOCs of champak flowers showed that over 85% of VOCs is constituted by terpenoids and volatile esters. Among the terpenoids, β-linalool and its derivatives (17.5%), β-ocimene (6.8%) and β-cubebene (8.6%) were the major compounds. However, we could not detect any volatile diterpenoids emitted from champak flowers suggesting that diterpenoids may not contribute to floral scent of champak. Among five monoterpenoids identified, β-linalool, β-ocimene and their derivatives were dominant accounting for more than 94% of total monoterpenoids. Both monoterpenes are not only known to be major components of floral fragrance [4, 40] but are also very common plant VOCs which are released after herbivore damage [41]. Furanoid and pyranoid linalool oxides are found as prominent constituents of the floral scent along with β-linalool in *Clarkia breweri* [42] and *Magnolia kobus* [43]. Further studies in *Arabidopsis* CYP76C1 mutants, which is a major linalool metabolizing oxygenase demonstrated linalool oxides as a repellent for a number of insect taxa [44]. Linalool metabolism might also serve as a detoxification mechanism of linalool, which is shown to be cytotoxic [45].

2-Methylbutanoate and its derivatives such as methyl 2-methylbutanoate, ethyl 2-methyl butanoate and propyl 2-methylbutanoate were some of the major components of champak floral VOCs. These are known to possess a fruity odor and contribute to flavors of strawberry and pineapple [46, 47]. Methyl 2-methylbutanoate was identified as the key volatile of *Magnolia ovata* in attracting cyclocephaline scarab beetles [48], suggesting that champak might be a cyclocephaline-pollinated flower. It is known that cyclocephaline and moth-pollinated flowers produce the attractant volatile in much larger quantities than the amount of attractant chemical produced by bee-pollinated flowers [49]. Our VOCs analysis suggests that champak flowers produce large quantities of 2-methylbutanoate derivatives to attract pollinators, at the same time, they convert linalool to linalool oxides to protect their reproductive tissues from insects.

Our metabolite-guided RNA-seq approach provided a framework to identify pathways and genes for the biosynthesis of floral VOCs from champak flowers. GPP is the universal precursor for all monoterpenes whose synthesis is catalyzed by gernayl diphosphate synthase. This enzyme can exist as homodimers or heterodimers depending on the plant species [50]. Our RNA-seq data indicates the GPS of champak is a heterodimer. GPS.SSU was one of the most abundantly expressed genes in champak flowers. The non-catalytic GPS.SSU is necessary to interact with the catalytic large subunit of GPS (GPS.LSU) to form the functional enzyme [50]. The unigene encoding the GPS.LSU was found to be expressed at lower levels when compared to the small subunit. Two transcripts encoding SAMT were among the top 20 highly expressed champak unigenes. SAMTs catalyze the transfer of methyl group from S-adenosyl-L-methionine (SAM) to various substrates and are essential for the formation of many volatile methyl esters such as methyl benzoate, methyl salicylate, and methyl jasmonate [51]. Although we could not identify methyl benzoate, methyl salicylate, or methyl jasmonate from VOCs of champak flowers, we were able to detect other volatile methyl esters such as 2-methylbutanoate, methyl 2-methylbutanoate, methyl tiglate, methyl hexanoate, methyl anthranilate, methyl palmitate, methyl linoleate and methyl linolenate (Fig. 1 and Table 1), all or some of which might be produced by the action of SAMTs. Not surprisingly, the genes encoding methionine synthase and SAM synthase that are crucial for biosynthesis of the methyl donor SAM were also among the most expressed unigenes. *Alcohol acyltransferase* (*AAT*) gene was also among the abundant transcripts, which catalyze the final step in the production of volatile esters by accepting a large range of alcohols and acyl-CoAs as substrates [52]. Therefore, AAT could be involved in the production of ethyl 2-methylbutanoate and propyl 2-methylbutanoate in champak flowers, respectively (Additional file 1: Figure S2).

Among the four DXS subfamily unigenes analyzed, McDXS1 and McDXS2 but not McDXS3 and McDXS4 encoded functional DXS enzymes when tested in a *dxs*-deficient *E. coli* strain. Members of DXS clade II are shown to play a significant role in secondary metabolites synthesis [53]. The two active DXS in champak, McDXS1 and McDXS2, belong to clade II suggesting that these 2 DXSs contribute towards the production of monoterpenes in champak flowers. McDXS3 and McDXS4 might catalyze a reaction distinct from that of McDXS1 and McDXS2. Since the amino acids essential for binding of G3P and TPP were conserved in McDXS4, it could catalyze a distinct reaction involving DXS substrates similar to CmDXS3/DXL from melon [54]. Unlike most plants that possess two isoforms of *HDR* [54], only single *HDR* (*McHDR*) was found from our champak RNA-seq (Additional file 1: Table S1). *McHDR* transcript level was more than 4 times higher in champak flowers than in leaves (Fig. 5a). This observation could either mean that champak has a single copy of *HDR* or the two isoforms may display spatial and temporal differential expression.

Linalool is one of the most common components of floral scent in a number of plant species [2]. Linalool synthases have been characterized from many plants [55–57]. Unlike many TPSs that form multiple products from a single substrate [4, 58], linalool synthases are likely to catalyze the formation of linalool exclusively [59]. The exception is the linalool synthase (PaLinS) from gymnosperm *Picea abies* (Norway spruce), which also produces very low quantities of nine monoterpenes including trans-β-ocimene, myrcene, α-terpinolene and 3-carene in vitro [58]. However, these minor compounds were not detected in transgenic tobacco plants expressing PaLinS [60].

Linalool synthases from multiple species do not share high amino acid sequence similarity. Owing to their substantially different sequences, linalool synthases from angiosperms were reported to belong to TPS-b, TPS-g and TPS-f subgroups of the TPS phylogenetic tree [61]. Even though McTPS1 belongs to TPS-b subgroup with the R(R)X_8_W motif for monoterpene cyclization and shares highest sequence similarity to trans-β-ocimene synthase from *Litsea cubeba*, it only produced an acyclic monoterpene alcohol, (R)-linalool in in vitro, in vivo assays and in transgenic plants. Linalool occurs as two enantiomers in nature: (R)-linalool in lavender and bay laurel [62]*,* whereas (S)-linalool is found in coriander [63] and *Clarkia breweri* [42]. (R)- and (S)-forms differ considerably in their olfactory qualities: (R)-form has a woody and lavender-like scent whereas the (S)-form as petitgrain-like and floral [64].

In addition to (R)-linalool, we found trans-furanoid and trans-pyranoid linalool oxides to contribute majorly to champak floral scent. Trans-furanoid linalool oxide was identified as the major product of biotransformation of (R)-linalool in *Aspergillus niger* [65]*.* Moreover, in fungus, biotransformation of linalool to furanoid and pyranoid linalool oxides was postulated to have epoxylinalool as an intermediate [66]. These results indicate that (R)-linalool could be converted into trans-furanoid and trans-pyranoid linalool oxides in champak flowers. The oxidation of (S)-linalool to epoxylinalool involved a single cytochrome P450 enzyme (CYP71B31) in *Arabidopsis* [67]. Additionally, cytochrome P450 76 family genes were also shown to be involved in linalool metabolism in *Arabidopsis* [44]. From our champak transcriptome data, we found few homologues of *Arabidopsis* cytochrome P450s involved in linalool metabolism. These would be the ideal candidates to analyze and identify the genes involved in the formation of furanoid and pyranoid linalool oxides.

## Conclusions

Availability of extensive genome resources for the commercially significant non-model plant *Magnolia champaca* is lacking. In this study, we performed de novo transcriptome assembly of high quality reads generated through Illumina paired end sequencing. Transcripts for the enzymes involved in biosynthesis of terpenes, phenylpropanoids/benzenoids and volatile esters, predominant VOCs in champak flowers were identified. Expression levels analysis of these unigenes showed that most of them were expressed higher in flowers than in leaves. Moreover, functions of the enzymes involved in the first and the last step of MEP pathway were validated by *E. coli* complementation assay. Finally, we also characterized the function of one of the most highly expressed terpene synthase as (R)-linalool synthase using in vitro, in vivo and transgenic studies. Our work will facilitate new gene discovery and provide crucial information for future genetic studies in champak. It will also serve as platform for metabolic engineering of champak floral-scent related genes.

## Methods

### Plant materials

Fully opened champak flowers were collected from the trees (10–12 m tall and 25–30 cm in diameter) grown in the National University of Singapore campus during the month of November for VOCs analysis and RNA-seq. Young leaves of about 10–12 cm length were collected for qRT-PCR analysis. All sample collection was carried out at 7:00–8:30 am. Fresh samples unattacked by herbivores/aphids were chosen for the study. The samples were used immediately for VOCs analysis using a push-pull headspace collection system and total RNA extraction. *Nicotiana benthamiana* plants were grown in greenhouse under long day condition (16 h L/8 h D) for 4 weeks before using them for in vivo assays and subcellular localization experiments. *Nicotiana tabacum* plants were used for *Agrobacterium*-mediated transformation [68].

### VOCs collection and analysis

For analysis of headspace chemical composition of champak flowers, floral VOCs were collected from 100 g of freshly collected flowers for 6 h in a tissue culture room at 25 °C using a push-pull headspace collection system [69]. Champak flowers were placed in a glass jar (15 cm diameter, 20 cm height; UFO Labglass, Singapore). A compressed air pump was used to pull headspace air through a sorbent trap filled with HayeSep-Q trap (80/100 mesh size; Restek, USA). One μl (100 μg/ml) of camphor was added as an internal standard to each sorbent trap. The floral VOCs along with camphor were extracted twice from sorbent traps with 200 μl of hexane and analyzed by Agilent GC 7890A with 5975C inert mass selective detector, equipped with a HP-5MS column (30 m × 0.25 mm, 0.25 μm film thickness; Agilent Technologies, USA). 5 μl sample was injected into the column heated to 250 °C and the temperature was increased from 50 °C (1 min hold) to 300 °C (1 min hold) at the rate of 8 °C min^−1^. Retention indices (RI) were calculated by using C_7_-C_30_ n-alkanes standard. The compounds were identified by comparison with mass spectra reference library NIST MS 2014 and by using RI match. The data were processed by MSD ChemStation Data Analysis (Agilent Technologies). The internal standard camphor was used in calculating the quantity of other compounds.

In order to determine the linalool enantiomer emitted by champak, gas chromatography (GC) was carried out with a CP-Chirasil Dex CB column (25 m × 0.25 mm, 0.25 μm film thickness; Agilent Technologies, USA) and mass spectrometry (MS) detector at 220 °C. The headspace extracts from champak flowers, in vitro and in vivo assay products were analyzed using split injection into the column heated to 250 °C and the temperature was increased from 40 °C (1 min hold) to 200 °C at the rate of 4 °C min^−1^. The enantiomeric identity of linalool was confirmed by comparison of GC data to authentic (±)-linalool and (R)-linalool standards.

### RNA isolation for RNA sequencing (RNA-seq)

Total RNA was isolated from homogenized flower sample using the Spectrum™ Plant Total RNA Kit (Sigma-Aldrich). The quantity and quality of RNA were measured by a Nanodrop spectrophotometer (ND-1000, Thermo Fisher Scientific) and Agilent 2100 Bioanalyzer and RNA 6000 Nano Labchip Kit (Agilent Technologies), respectively. RNA sample with RNA Integrity Number (RIN) of >7 was sent to the Rockefeller University Genomics Resource Center (New York, USA) to carry out next generation sequencing using Illumina HiSeq 2000. De novo assembly of the transcripts were performed as described in [70].

### cDNA synthesis and quantitative real time PCR (qRT-PCR)

1 μg of total RNA was used for cDNA synthesis with M-MLV reverse transcriptase (Promega), dNTP and oligo dT. The resulting 25 μl of RT reaction product was diluted to a total volume of 100 μl with RNase-free water and stored at −20 °C until use.

The expression levels of selected genes were analyzed by qRT-PCR. The cDNA sequences obtained from the RNA-seq data were exploited for designing primers using the Primer3 program [71]. The primers used in this study are listed in Additional file 1: Table S4. The PCR reaction mixtures were subjected to the following conditions: (50 °C for 2 min, 95 °C for 10 min; 45 cycles of 95 °C for 15 s and 60 °C for 1 min) in the Applied Biosystems 7900HT fast real-time PCR system. *Actin* amplification was used as an internal normalization. Non-template control and non-RTase treated templates were included for each gene in order to eliminate the possibility of primer dimer formation and random genomic DNA contamination. All qRT-PCR experiments were carried out in triplicates with biological replicates. The results obtained were analyzed using SDS 2.4 software (Applied Biosystems).

### Phylogenetic analysis

The deduced amino acid sequence of McDXSs and McTPS1 were aligned with DXSs and TPSs from other plant species using Clustal W under the following parameters: gap open - 3; gap extension - 1.8; Gonnet; penalties - on; gap separation - 4; cut off - 30%. Best-fit maximum likelihood models were identified for the alignment files using MEGA 6.0 software. The maximum likelihood trees for McDXS and McTPS1 were constructed with JTT + G5 and LG + G5 models respectively. 1000 bootstrap replications were made for each of the above trees.

### Vector construction for *Agrobacterium*-mediated gene expression

Full-length ORFs of *McDXS1–4*, *McHDR* and *McTPS1* were amplified from champak flower cDNAs by PCR. Gene specific primers were based on cDNA sequences of *McDXS1–4*, *McHDR* and *McTPS1* from champak RNA-seq data. The purified PCR products were cloned into pDONR221 vector using BP clonase (Invitrogen). All constructs were verified by DNA sequencing.

In order to generate YFP fusion constructs, pDONR221 containing *McDXS1*, *McDXS2*, *McDXS3*, *McDXS4*, *McHDR* and *McTPS1* were integrated into pBA-DC-YFP expression vector, which contains the cauliflower mosaic virus (CaMV) 35S promoter using LR clonase (Invitrogen).

### Subcellular localization of McDXSs, McHDR and McTPS1

For subcellular localization, plasmids harbouring *McDXS1-YFP*, *McDXS2-YFP, McDXS3-YFP, McDXS4-YFP, McHDR-YFP* and *McTPS1-YFP* constructs were transformed into *Agrobacterium tumefaciens* GV3101 strain. The transformed GV3101 cells were then spread on LB plates with spectinomycin (100 μg/ml) and gentamycin (20 μg/ml) and incubated at 28 °C for two days. Cultures obtained from single colony of the above plates were infiltrated into the leaves of 4 weeks old *N. benthamiana* plants using a 1 ml syringe. The infiltrated leaves were excised three days post infiltration (dpi) and observed under a confocal laser-scanning microscope (Carl Zeiss LSM 5 Exciter) with a standard filter set. Images were analyzed by the Carl Zeiss’s LSM image browser.

### Functional complementation assays using *Escherichia coli* mutants

Full-length ORFs of *McDXS1*, *McDXS2*, *McDXS3*, *McDXS4, MsHDR* along with *Arabidopsis DXS1* and *HDR* were inserted into gateway destination vector pDEST17 (Thermo Fisher Scientific) for expression in *E. coli*. Constructs containing *DXS* and *HDR* genes were transformed into *E. coli dxs* ^−^ strain, defective in DXS activity [36] and *E. coli* HDR mutant strain MD1655 *ara*<>*ispH* [37], respectively.

For DXS complementation assay, the transformed cells were grown overnight at 37 °C on LB agar plates containing 1 mM mevalonate and ampicillin (100 μg.ml^−1^). Colonies were then transferred to LB agar-ampicillin plates lacking mevalonate and incubated at 37 °C overnight. *E. coli dxs* ^−^ strain transformed with *AtDXS1* and empty pDEST17 vector served as positive and negative controls, respectively.

HDR complementation assay was done with same procedure as above except media composition. The transformants were selected on LB agar plates containing 0.2% (*w*/*v*) arabinose, ampicillin (100 μg.ml^−1^) and kanamycin (50 μg.ml^−1^), then shifted to LB agar-ampicillin/kanamycin plates containing 0.2% (*w*/*v*) glucose instead of arabinose to suppress the expression of endogenous *HDR* and incubated at 37 °C overnight. *AtHDR* and *McDXS1* were used as positive and negative controls, respectively.

### Preparation of recombinant proteins

To construct vector for the recombinant N-terminal 6His-tagged protein, pDONR221 clone possessing the ORF of *McTPS1* was integrated into the destination vector, pDEST17 to generate pDEST-McTPS1. The final construct was transformed into *E. coli* BL21 pLysS strain. *E. coli* extract after isopropyl β-D-1-thiogalactopyranoside (IPTG) induction was incubated with Ni-NTA Sepharose resin (Qiagen). The bound proteins were then eluted using 250 mM imidazole.

### In vitro and in vivo TPS assay

In vitro TPS assay was carried out by mixing 250 μl of 2X reaction buffer (50 mM HEPES pH 7.4, 200 mM KCl, 15 mM MgCl_2_, 10% glycerol, 10 mM DTT) with 20 μg purified recombinant protein and 10 μg of substrate (GPP and FPP) in an inert glass bottle. The reaction was mixed well and overlaid slowly with 250 μl of hexane. The reaction bottle was tightly closed and sealed with parafilm before incubating at 30 °C for 2 h. After 2 h incubation, the reaction mixture was vortexed for 1 min and centrifuged at 1200 rpm for 30 min. The hexane layer was then transferred to a fresh glass GC bottle (Agilent Technologies) and subjected to GC-MS (see VOCs collection and analysis).

In vivo characterization of TPS using an *Agrobacterium*-mediated transient assay in *N. benthamiana* was performed according to Jin et al., (2015) with exception to the VOCs collection procedure. VOCs were collected and analyzed from four *N. benthamiana* plants infiltrated either with McTPS1-GFP or GFP using headspace method as described in VOCs collection and analysis. The compound obtained was identified with the authentic (±)-linalool standard (Sigma-Aldrich) and by mass spectra reference library.

## Additional file 1

Additional file 1: Figure S1. Quality and KEGG analysis of champak RNA-seq. **Figure S2.** Proposed biosynthesis pathways of volatile ester 2-methylbutanoate and its derivatives via the catabolism of branched-chain amino acid L-isoleucine. **Figure S3.** Comparison of deduced amino acid sequence of representative genes from pathways responsible for the production of VOCs. **Figure S4.** Phylogenetic analysis and amino acid alignment of champak DXSs. **Figure S5.** Amino acid sequence alignment of McHDR. **Figure S6.** Amino acid sequence alignment of McTPS1. **Figure S7.** GC-MS chiral analysis of β-linalool emitted from champak flowers. **Figure S8.** Analysis of transgenic *N. tabacum* overexpressing *McTPS1.* **Table S1.** MEP pathway genes from champak RNA-seq. **Table S2.** TPS genes from champak RNA-seq.** Table S3.** Accession numbers of proteins used in the TPS phylogenetic analysis. **Table S4.** List of primers used in this study. **Table S5.** Accession numbers of proteins used in the amino acid sequence alignments. **Table S6.** Accession numbers of proteins used in the DXS phylogenetic analysis. (PDF 1708 kb)
Additional file 2: Functional annotation of champak unigenes using BLASTX against the multiple protein databases. (XLS 13237 kb)

## Abbreviations

CaMV: Cauliflower mosaic virus
CYP450: Cytochrome P450s
FAME: Fatty acid-derived methyl esters
FPKM: Fragments per kilobase of transcript per million mapped reads
GC-MS: Gas chromatography-mass spectrometry
GFP: Green fluorescent protein
GO: Gene ontology
IPTG: Isopropyl β-D-1-thiogalactopyranoside
KAAS: KEGG automatic annotation server
KEGG: Kyoto encyclopedia of genes and genomes
KO: KEGG orthology
MEP: Methylerythritol 4-phosphate
MVA: Mevalonate
NCBI: National centre for biotechnology information
NR: Non-redundant
ORF: Open reading frame
qRT-PCR: Quantitative real-time polymerase chain reaction
RNA-seq: RNA sequencing
TP: Transit peptide
TPS: Terpene synthase
VOC: Volatile organic compound
WT: Wild type
YFP: Yellow fluorescent protein
