# Supplementary material for: Integrated metabolome and transcriptome analysis of Magnolia champaca identifies biosynthetic pathways for floral volatile organic compounds
Source: BMC Genomics. 2017 Jun 14;18:463. doi: 10.1186/s12864-017-3846-8 (PMC5471912; doi:10.1186/s12864-017-3846-8)
Supplement: Additional file 1: Figure S1. — Quality and KEGG analysis of champak RNA-seq. Figure S2. Proposed biosynthesis pathways of volatile ester 2-methylbutanoate and its derivatives via the catabolism of branched-chain amino acid L-isoleucine. Figure S3. Comparison of deduced amino acid sequence of representative genes from pathways responsible for the production of VOCs. Figure S4. Phylogenetic analysis and amino acid alignment of champak DXSs. Figure S5. Amino acid sequence alignment of McHDR. Figure S6. Amino acid sequence alignment of McTPS1. Figure S7. GC-MS chiral analysis of β-linalool emitted from champak flowers. Figure S8. Analysis of transgenic N. tabacum overexpressing McTPS1. Table S1. MEP pathway genes from champak RNA-seq. Table S2. TPS genes from champak RNA-seq. Table S3. Accession numbers of proteins used in the TPS phylogenetic analysis. Table S4. List of primers used in this study. Table S5. Accession numbers of proteins used in the amino acid sequence alignments. Table S6. Accession numbers of proteins used in the DXS phylogenetic analysis. (PDF 1708 kb) [file 12864_2017_3846_MOESM1_ESM.pdf]

Additional file 1

a

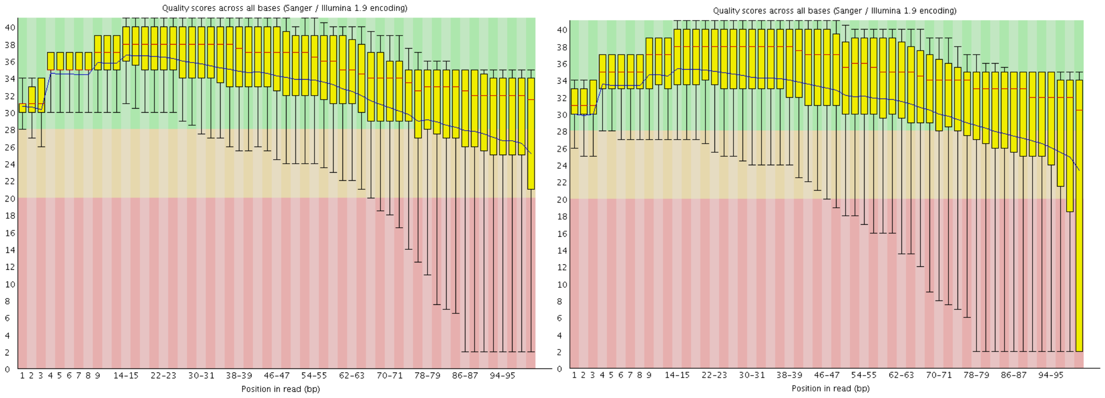

b

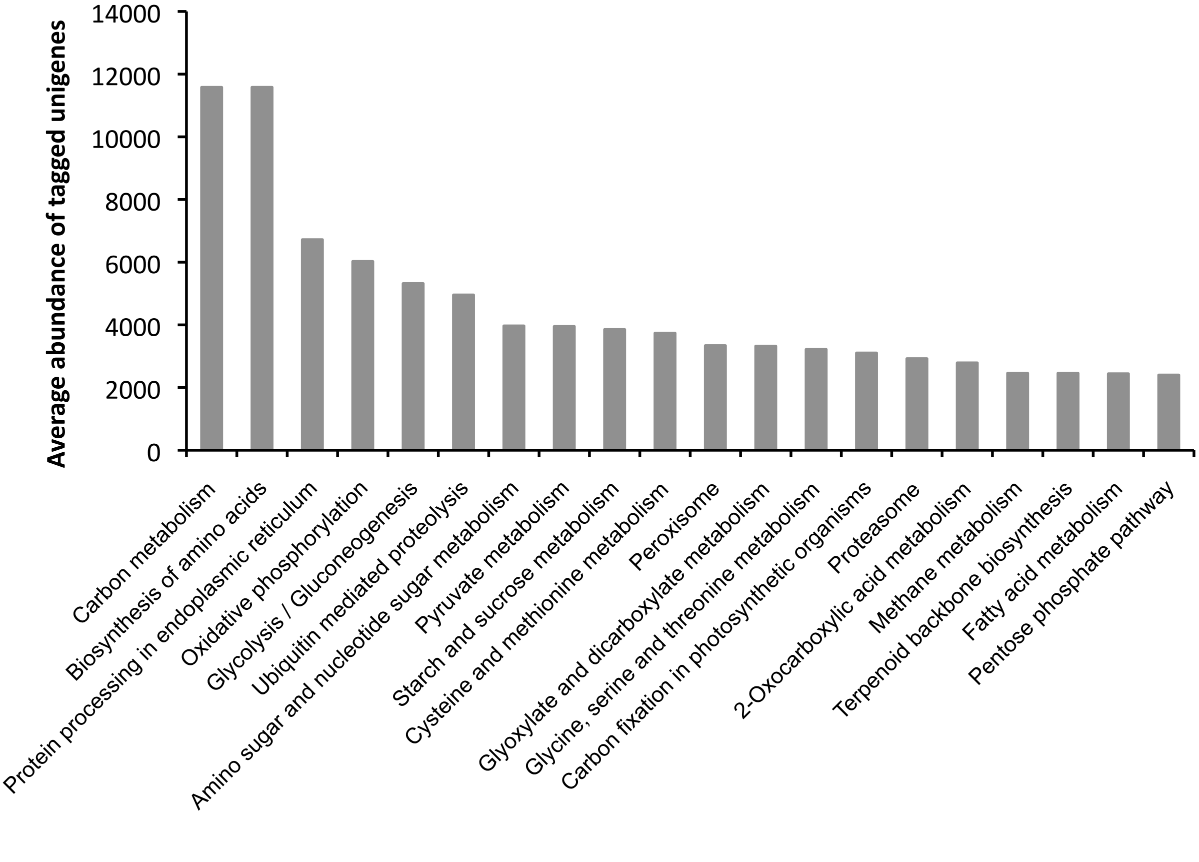

Figure S1. Quality and KEGG analysis of champak RNA-seq. (a) The overview of sequence quality was generated using the FastQC software. Phred quality scores vs read position for 2*101 bp paired end sequencing of champak flower RNA libraries on an Illumina HiSeq2000. The green area indicate good quality, yellow is reasonable quality and red is poor quality. The blue line indicate the mean score and the box plots represent the score spread. (b) Top twenty KEGG pathways identified from champak flower transcriptome data.

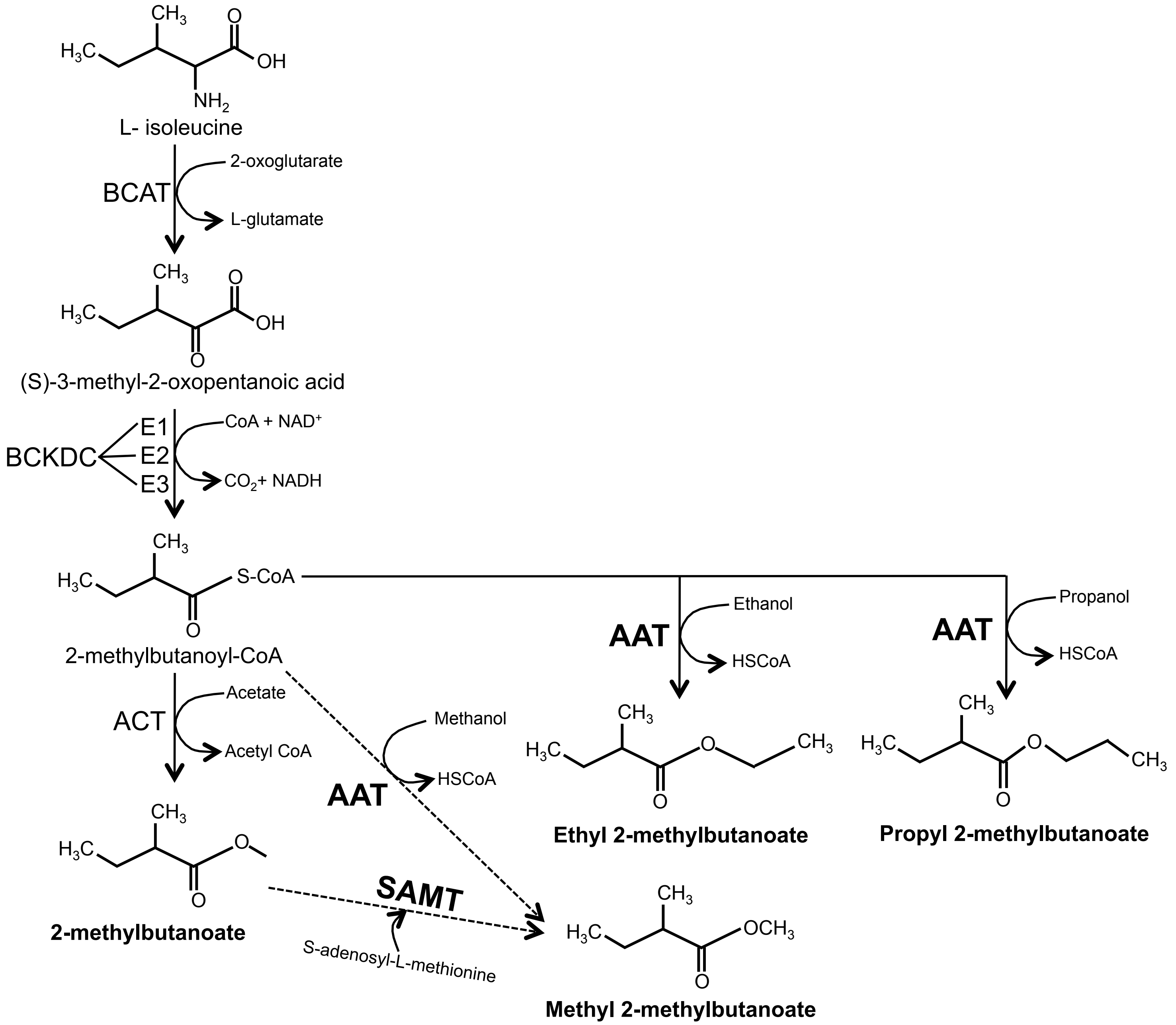

Figure S2. Proposed biosynthesis pathways of volatile ester 2-methylbutanoate and its derivatives via the catabolism of branched-chain amino acid L-isoleucine. Solid arrows indicate proven steps by previous studies [1-3] while the dotted arrows indicate proposed steps in the biosynthesis pathway. BCAT: branched-chain amino acid aminotransferase; BCKDC: branched-chain α-ketoacid dehydrogenase complex; E1: pyruvate dehydrogenase; E2: lipoamide acyltransferase, E3: dihydrolipoamide dehydrogenase; ACT: acetate-CoA transferase; AAT: alcohol acyl transferase; SAMT: S-adenosyl-L-methionine dependent methyltransferase.

References

1. Islam MM, Nautiyal M, Wynn RM, Mobley JA, Chuang DT, Hutson SM. Branched-chain amino acid metabolon: interaction of glutamate dehydrogenase with the mitochondrial branched-chain aminotransferase (BCATm). J Biol Chem. 2010;285:265-276.
2. Perez AG, Olias R, Luaces P, Sanz C. Biosynthesis of strawberry aroma compounds through amino acid metabolism. J Agric Food Chem. 2002;50:4037-4042.
3. Tressl R, Albrecht W. Biogenesis of aroma compounds through acyl pathways. In: Parliment TH, Croteau R, editors. Biogeneration of Aromas. Washington, DC: American Chemical Society; 1986. p. 114-133.

a

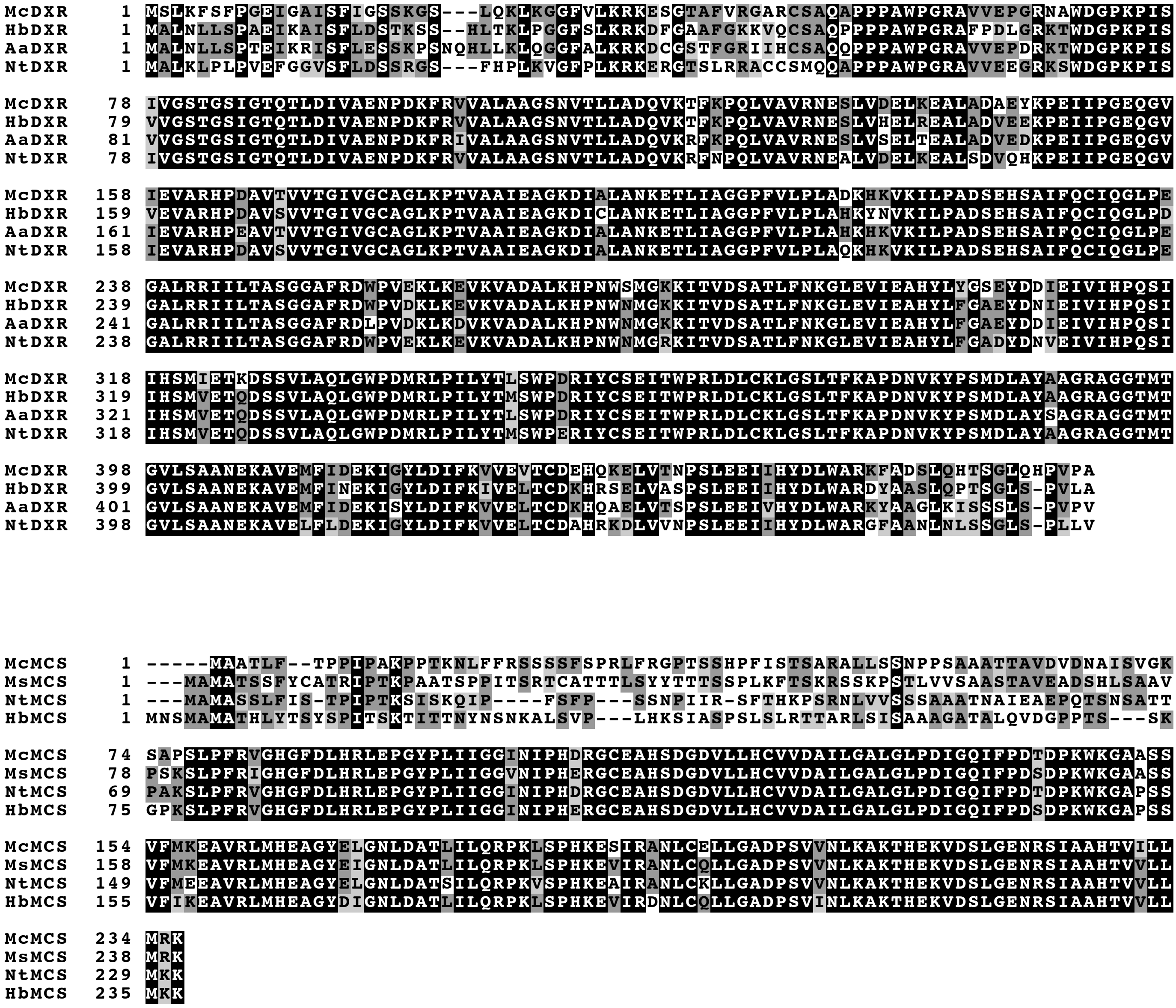

Figure S3. Comparison of deduced amino acid sequence of representative genes from pathways responsible for the production of VOCs. a. Multiple sequence alignment of deduced amino acid sequences of two representatives, 1-deoxy-D-xylulose 5-phosphate reductoisomerase (DXR) and 2-C-methyl-D-erythritol 2,4-cyclodiphosphate synthase (MCS), from MEP pathway. Accession numbers of proteins used in the sequence alignment analysis are given in Additional file 1: Table S5. The amino acid sequences were aligned using CLUSTALW. Completely conserved amino acids are shown in white with black background. Residues conserved in three sequences are shown in black with dark grey background.

b

```
McAACT    1 MAP--AAASDSIKPRDVCVVGVARTPMGGFLGTLSSLSATKLGSIAIECALKRAGVDPKLVQEVYFGNVLSANLGQAPAR
TcAACT    1 MAPTAAAASDSIKPRDVCVVGVARTPMGGFLGSLSSLSATKLGSIAIEAALKRANVDPSLVQEVIFGNVLSANLGQAPAR
PtAACT    1 -----MASSDSIKPRDVCIVGVARTPMGGFLGSLSSFSATKLGSIAIQCALQRANIDPSLVQEVFFGNVLSANLGQAPAR
HbAACT    1 ----MSPSSDSINPRDVCIVGVARTPMGGFLGSLSSFSATKLGSIAIQAAIKRANVDPSLVQEVFFGNVLSANLGQAPAR

McAACT   79 QAALGAGIPNTVICTTINKVCASGMKATMFAAQSIQLGINDVVVAGGMESMSNAPKYLSEARKGSRLGHDTVVDGMLKDG
TcAACT   81 QAALGAGIPNSVICTTVNKVCASGMKATMLAAQSIQLGINDVVVAGGMESMSNAPKYLAEARKGSRLGHDTLVDGMMKDG
PtAACT   76 QAALGAGIPNSVICTTINKVCASGMKATMLAAQTIQLGINDVVVAGGMESMSNAPKYLADARKGSRLGHDTIVDGMMKDG
HbAACT   77 QAALGAGIPNSVICTTINKVCASGMKATMLAALTIQAGINDIVVAGGMESMSNAPKYLAEARRGSRLGHDTITDGMLKDG

McAACT  159 LWDVYNDYGMGMCAELCADQHMITREEQDSYGIQSFERGIAARNGGAFAWEIVPVEVSGGRGKPSVLVDNDEGLEKFDPV
TcAACT  161 LWDVYNDCGMGSCAELCAEKHVFTREDQDNFSIQSFERGIAAQQGGAFAWEIVPVEVSGGRGKPSTIVDKDEGLGKFDAA
PtAACT  156 LWDIYNDFGMGVCAEICADQHSITRDDQDSYAIQSFERGIAAQNSGHLSWEVVPVEVSGGRGKPFTIVDKDDGLGKFDAA
HbAACT  157 LWDVYNDFGMGVCAEICADQHNITREEQDSYAIRSFERGNSAQNGGVFSWEIVPVEVSGGRGKSVMVVDKDEGLIKFDAA

McAACT  239 KLRKLRPNFKENGGSVTAGNASSISDGAAALVLVSGEKALELGLQVIAKISGYADAAQAPELFTTTPALAIPKAISNAGL
TcAACT  241 KLRKLRPSFKDNGGTVTAGNASSISDGAAALVLVSGEKALKLGLQVIAKITGYADAAQAPEFFTTAPALAIPKAISNAGL
PtAACT  236 KLRKLRPSFKENGGSVTAGNASSISDGAAALVLMSGEKALKLGLQVIAKIRGYADAAQAPELFTTAPALAIPKAISNAGL
HbAACT  237 KLRKLRPSFK-NGGSVTAGNASIISDGAAALVLVSGEKAIELGLQVIARIRGYGDAAQAPELFTTAPALAIPKAISNAGL

McAACT  319 EASQIDFYEINEAFAVVAVANQKLLGIHPDKLNVHGGAVSLGHPLGCSGARILVTLLGVLRQRNGKYGAAGICNGGGGAS
TcAACT  321 DASQIDYYEINEAFAVVALANQKLLDLNPEKVNVNGGAVSLGHPLGCSGARILVTLLGVLKQRNGKYGVGGVCNGGGGAS
PtAACT  316 EASQIDFYEINEAFSVVALANQKLLGLNPQKVNAHGGAVSLGHPLGCSGARILVTLLGVLKHKNGKYGVGGICNGGGGAS
HbAACT  316 EASQIDYYEINEAFSVVALANQKILGLNPEKLNVHGGAVSLGHPLGCSGARILVTLLGVLRHKNGKYGVASICNGGGGAS

McAACT  399 ALVLELMPVIRAERSLL
TcAACT  401 ALVVELL----------
PtAACT  396 ALVLELMQVARVGPSSL
HbAACT  396 ALVLELMSVGKVGRSLL
```

```
McMVK     1 MEVRTRAPGKIILSGEHAVVHGSTAVAGSIDLYTNVSLRISNSSDVDDGLLEFRLKDMGLAFSWKIQKIKETISELGFPL
MnMVK     1 MEVKARAPGKIILSGEHAVVHGSTAVAASIDLYTYVTLRFPTPSDDDD-NLKLQLKDMALEFSWPIGRIKEELPGLGCPD
HbMVK     1 MEVKARAPGKIILSGEHAVVHGSTAVAASINLYTYVTLSFAT-AENDD-SLKLQLKDMALEFSWPIGRIREALSNLGAPP
PnMVK     1 MEVRARAPGKIILAGEHAVVHGSTAVAASIDLYTYASLHFPSPSDNDN-TLKLQLKDLDLEFSWTVQRIKDAFCDRGGCN

McMVK    81 SSSAPSFSSESIKLFATLVEQQNIPEAKIGLAAGISAFLWLYTSIQGFKPATAVVTSDLPLGSGLGSSAAFCVSISAALL
MnMVK    80 SSIATSCSIEAAKVLAALVEEQNIPEAKITLASGVSAFLWLYTSIQGYKPATIVVTSELPIGSGLGSSAAFCVALSAALL
HbMVK    79 SSTRTSCSMESIKTISALVEEENIPEAKIALTSGVSAFLWLYTSIQGFKPATVVVTSDLPLGSGLGSSAAFCVALSAALL
PnMVK    80 ASSPTSCSPETIKIIAALVEEQYIPEAKIGLAAGVTAFLWLYTSIQGYKPAKVIVTSELPLGSGLGSSAAFCVSLSAAFI

McMVK   161 ASSGALSSVSSHDGWLTLGESELELVNRWAFEGEKIIHGKPSGIDNTVSTFGNMIMFKSGKLTRIKPSMPLRMLVTNTKV
MnMVK   160 AFSDLVKIDLSHQGWVMFAEDELDLLNKWAFEGEKIIHGRPSGIDNTVSTYGNLIKFRSGSLTRIKSNLPLKMLITNTKV
HbMVK   159 AFSDSVNVDTKHQGWSIFGESDLELLNKWALEGEKIIHGKPSGIDNTVSTYGNMIKFKSGNMTRIKSNMPLKMLVTNTRV
PnMVK   160 ALSDSVNLDFNHQGWLMFGESKLELVNKWAFEGEKLIHGKPSGIDNTVSTFGNMIKFRSGALTRMKSNMQLKMLITNTKV

McMVK   241 GRNTKALVAGVSERACRHPEAMTAVFTAVDSISKELAAIIQSPASDMLAITEKEDKLEELMEMNQGLLQSMGVSHSSIET
MnMVK   240 GRNTKALVAGVSERTLRHANAMSFVFNAVDSISEELASIIQSPIGDDLSVPQKEEKIEELMEMNQGLLQCMGVSHASIET
HbMVK   239 GRNTKALVAGVSERTLRHPNAMSFVFNAVDSISNELANIIQSPAPDDVSITEKEEKLEELMEMNQGLLQCMGVSHASIET
PnMVK   240 GRNTKALVASVSERTFRHPDAMTAVFTAVDSISNKLATIIESPASDECAITEKEVLVEELMEMNQGLLQCMGVSHASIET

McMVK   321 VLRTTLKYKLASKLTGAGGGGCVLTLLPALLSKTIVDKVIAELESCGFQCLIAAIGGKGIEIGFCGYS
MnMVK   320 VLRTTLKYKLASKLTGAGGGGCVLTLLPNLLSGTIADRVTSDLESSGFQCFTAGIGGNGVEIYLGGSS
HbMVK   319 VLRTTLKYKLASKLTGAGGGGCVLTLLPTLLSGTVVDKAIAELESCGFQCLIAGIGGNGVEFCFGGSS
PnMVK   320 VIRTTLKYKLATKLTGAGGGGCVLTLLPTILSGKIVDNVIAELESCGFQCLIAGIGGNGLEICFGCSA
```

Figure S3. (Continued). b. Multiple sequence alignment of deduced amino acid sequences of two representatives, acetyl-CoA acetyltransferase (AACT) and mevalonate kinase (MVK), from MVA pathway.

c

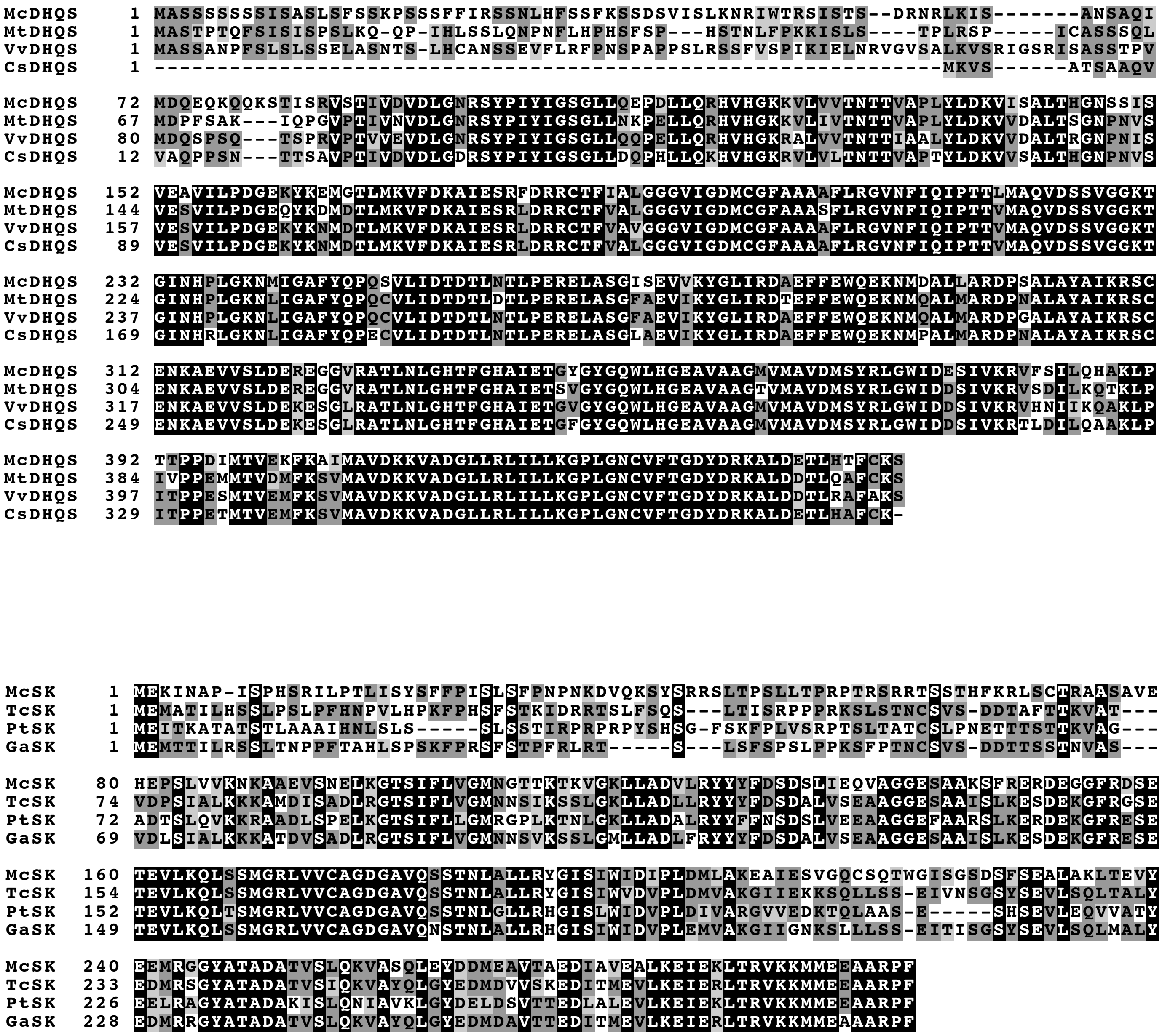

Figure S3. (Continued). c. Multiple sequence alignment of deduced amino acid sequences of two representatives, 3-dehydroquinate synthase (DHQS) and shikimate kinase (SK), from shikimate pathway.

d

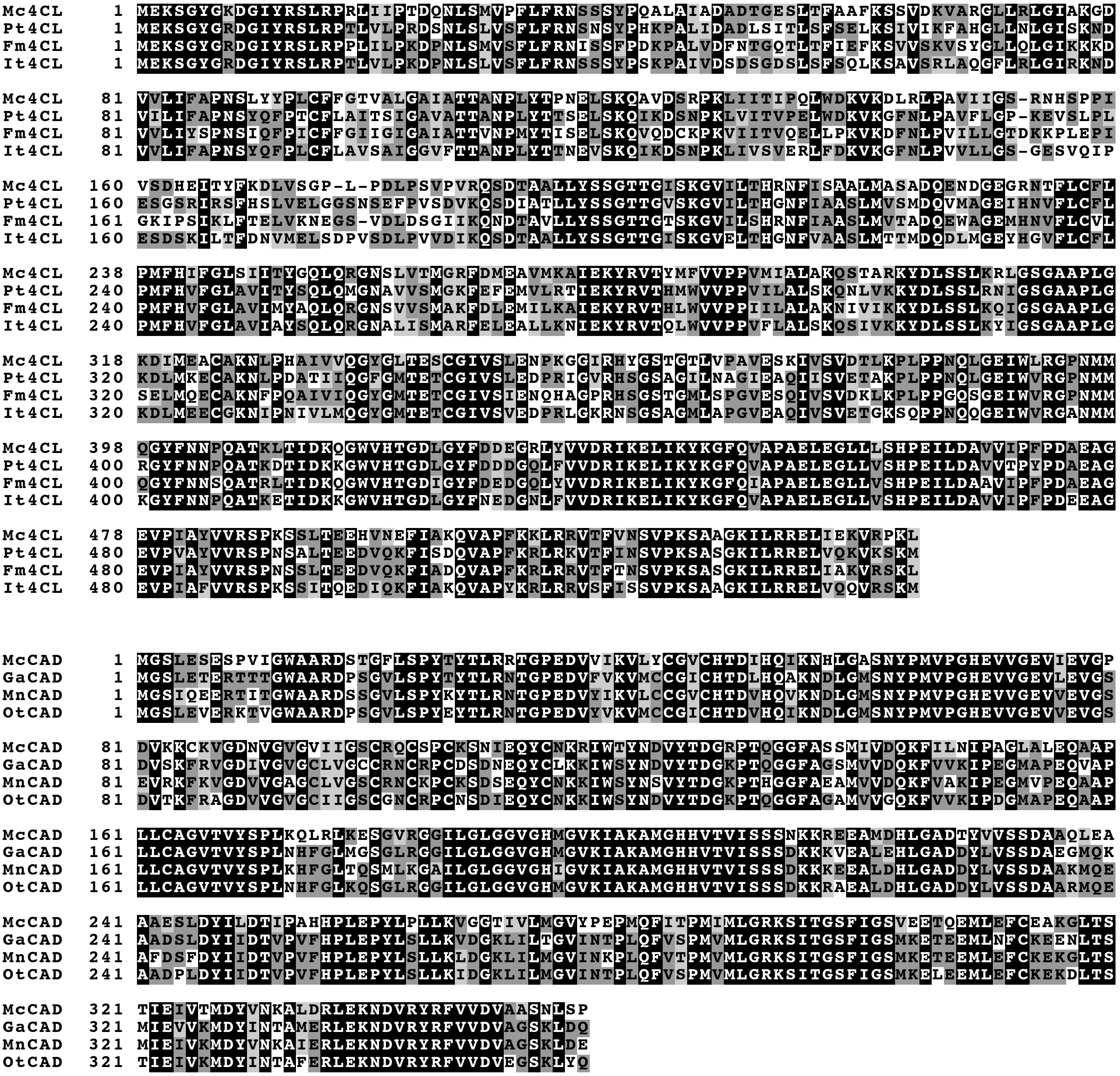

Figure S3. (Continued). d. Multiple sequence alignment of deduced amino acid sequences of two representatives, 4-coumaroyl-CoA ligase (4CL) and cinnamyl alcohol dehydrogenase (CAD), from phenylpropanoid/benzenoid pathway.

e

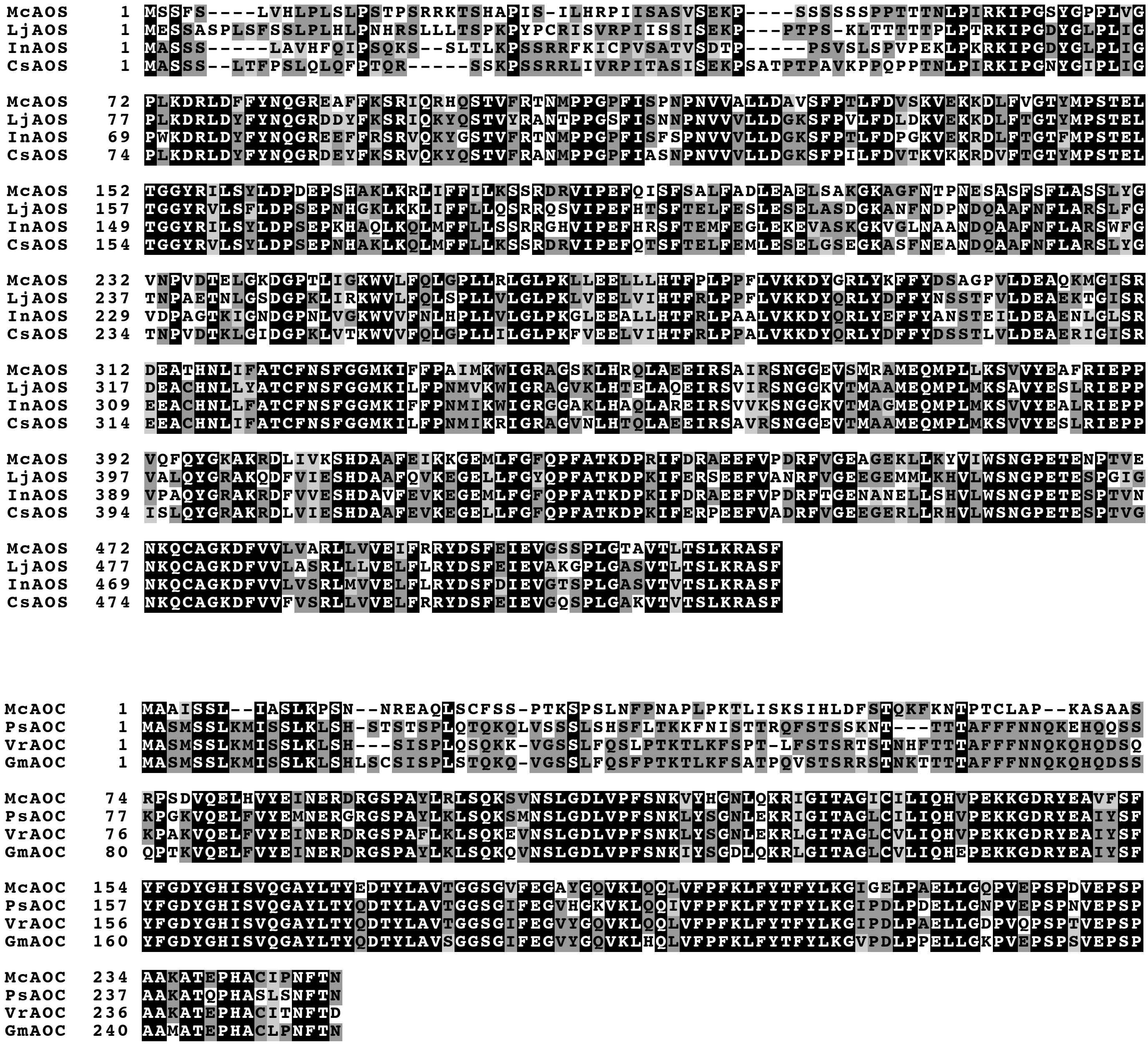

Figure S3. (Continued). e. Multiple sequence alignment of deduced amino acid sequences of two representatives, allene oxide synthase (AOS) and allene oxide cyclase (AOC), from lipoxygenase pathway.

f

```
McBCAT    1 MFLRRLCCCDTIKFSEKLLDRYR---------FTKFGNYR-------------FYTSRAASS-------SQQIASDIYR-
GaBCAT    1 --MESNAVLATLQPNYNILSPSRFYSSSSSAATTFLSVFYPQRTRF--SPPQYLKLDKQVLAFASCRYAHEVKSPFKNA
RcBCAT    1 --METSAVLGSLHTNY-LISPSR--------NASFLLPHRPLHFSPS--SVPLSFKVRKQNPSYSSSNASLQAVSPFSRD
CsBCAT    1 --MESTAALTSLRPNYLLCSSRR---------FSPFLHSSSSSTSSFNHSSPLFLKKKLRLPSIN----APQTASPIS--

McBCAT   51 -------SDEDEVN-VNWEELGFGIVPTDYMYVMKCSKEDRFSEGKLNRFGNIEMSPSAGVLNYGQGLFEGLKAYKRED
GaBCAT   77 VLSDSYSSEASELADIEWDNLGFGLLPTDYMYMMKCSQGGNFSKGELQRFGNIELNPSAGVLNYGQGLFEGLKAYRKED
RcBCAT   69 IVSDTYGSQTTELADIDWDNLGFAYVPTDYMYIMKCVRDGSFSSGGLQRFGNIELSPSAGVLNYGQGLFEGLKAYRKED
CsBCAT   64 -------KDVGAIPEFDWDNLGFGIIPTDYMYVMKCSQGDNFSNGELKRFGNIELSPSAGVLNYGQGLFEGLKAYRKED

McBCAT  123 CLALFRPEENALRMQKGAERMCMPSPSVGQFVDAVKQTVLANKRWVPPPGKGSLYVRPLLIGSGPVLGLAPAPEYTFLI
GaBCAT  157 NILLFRPEENALRMRQGAERMCMPAPTVDQFVEAVKETVLANKRWVPPPGKGSLYIRPLLMGSGAVLGLAPAPEYTFLI
RcBCAT  149 NILLFRPEENALRMKIGAERMCMPSPTVEQFVEAVKATVLANKRWVPPPGKGSLYIRPLLMGSGAVLGLAPAPEYTFLI
CsBCAT  137 SILLFRPEENALRMKMGAERMCMQSPTVDQFVEAVKTTVLANKRWVPPSGKGSLYIRPLLMGSGAVLGLAPAPEYTFVI

McBCAT  203 ASPVGNYFKEGLSPINLFVDDEFHRATPGGTGSVKTIGNYAPVLKAQALAKSKGFSDVLYLDSINKRNLEEASSCNVFI
GaBCAT  237 VSPVGNYFKEGVAPINLIVEHELHRATPGGTGGVKTIGNYAAVLKAQSAAKAKGYSDVLYLDCVHKKYLEEVSSCNIFV
RcBCAT  229 VSPVGNYFKEGVAAINLIVEHELHRATPGGTGSVKTIGNYAAVLKAQSVAKAKGYSDVLYLDCVHKKYLEEVSSCNIFI
CsBCAT  217 VSPVGNYFKEGLAPINLVVEHELHRATPGGTGSVKTIGNYAAVLKAQSAAKAKGYSDVLYLDCVHKKYLEEVSSCNIFV

McBCAT  283 KNDVISTPAIEGTILPGITRKSIIEICPHYGYQIQERPVSVEELMDADEVFCTGTAVVIAPVGSIMYHGKRVEYR-IGE
GaBCAT  317 KGDVISTPAIKGTILPGITRKSIIDVARSQGFQVEERFVPVDELLDADEVFCTGTAVVVSPVGSITYKGKRVSYGVDGF
RcBCAT  309 KDKVISTPAVKGTILPGITRKSIIDVARSQGFQVEERPVAVDELLDADEVFCTGTAVVVSPVGSITYMGRRVSYGESGF
CsBCAT  297 KGNVISTPAIKGTILPGITRKSIIDVARSLGFQVEERLVAVDELLEADEVFCTGTAVVISPVGSVTYLGKRIAYG-NGV

McBCAT  362 TVSQKFYSALTSIQMGLVEDKMGWTVEID--
GaBCAT  397 AVAQQLYSVLTRLQMGLIDDKMDWTVELRIL
RcBCAT  389 AVSQQLYTVLTRLQMGLTEDRMNWTIELN--
CsBCAT  376 VVSQQLYSVLTRLQMGLIEDKMNWTVSLS--


McAAT     1 ---MASSDLVFSVTRGKPVLVSPAKPTPHEFKLLSDIDDQECLRFHMPILQFYRNDPSMRGRDPVGVIRDAIAKALVFYY
CmAAT     1 ----MASSLVFQVQRSQPQLIPPSDPTPHEFKQLSDIDDQEGLRFQIPVIQFYRHDPRMAGTDPARVIKEAIAKALVFYY
NtAAT     1 MDSKQSSELVFTVRRQKPELIAPAKPTPREIKFLSDIDDQEGLRFQIPVIQFYHKDSSMGRKDPVKVIKKAIAETLVFYY
PhAAT     1 MDSKQSSELVFTVRRQEPELIAPAKPTPRETKFLSDIDDQEGLRFQIPVINFYRKDSSMGGKDPVEVIKKAIAETLVFYY

McAAT    78 PFAGRIREGPGRKLTVECTGEGVVFIEADADVSLKQFGDVLHPPFPCLEKLLYDVPGMGEIINCPLLLIQVTRLTCGGFI
CmAAT    77 PFAGRLREGPGRKLFVECTGEGVMFIEADADVSLEQFGDALQPPFPCLEEPLFDVPNSSGVLDCPLLLIQVTRLKCGGFI
NtAAT    81 PFAGRLREGNGRKLMVDCTGEGIMFVEADADVTLEQFGDELQPPFPCLEELLYDVPDSAGVLNCPLLLIQVTRLRCGGFI
PhAAT    81 PFAGRLREGNDRKLMVDCTGEGVMFVEANADVTLEEFGDELQPPFPCLEELLYDVPGSAGVLHCPLLLIQVTRLRCGGFI

McAAT   158 FALSLNHTMCDGTGIVRFMSAVGELARGARAPSVLPSWGRERLSARNPPRVTHVHRQYDQVTDTKSNVTPLNEMAYRSFT
CmAAT   157 FALRLNHTMSDASGLVQFMMAVGEMARGATAPSVRPVWQRALLNARDPPKVTCHHREYDEVVDTKGTIIPLDDMAHRSFF
NtAAT   161 FALRLNHTMSDAPGLVQFMTAVGEMARGASAPSILPVWCRELLNARNPPQVTCTHHEYDEVRDTKGTIIPLDDMVHKSFF
PhAAT   161 FALRLNHTMSDAPGLVQFMTAVGEMARGATAPSTLPVWCRELLNARNPPQVTCTHHEYEEVPDTKGTLIPLDDMVHRSFF

McAAT   238 FGPREISILRKQVPHHLRTCSSFELLSGFFWRCRTIAVQPAPDEEMRYIFFFNVRDKCQPPLPAGYYGNG-ALAAAISPA
CmAAT   237 FGPSEISAIRKALPSHLRQCSSFEVLTACLWRFRTISLQPDPEEEVRVLCIVNSRSKFNPPLPTGYYGNAFAFPVALTTA
NtAAT   241 FGPSEVSALRRFVPHHLRKCSTFELLTAVLWRCRTMSLKPDPEEEVRALCIVNARSRFNPPLPTGYYGNAFAFPVAVTTA
PhAAT   241 FGPTEVSALRRFVPPHLHNCSTFEVLTAALWRCRTISIKPDPEEEVRVLCIVNARSRFNPQLPSGYYGNAFAFPVAVTTA

McAAT   317 SKLCQNPLAYAVELVKKAKSDVTQEYIQSIADLMVIKGRPCITLPGTYIVSNVTRLAFTDVDFGWGKAVYGGPARGIVYD
CmAAT   317 GKLCQNPLGYALELVRKAKADVTEDYMKSVADLMVIKGRPHFTVVRTYLVSDVTRAGFEDVDFGWGKAMYGGPAKGGVG-
NtAAT   321 AKLSKNPLGYALELVKKTKSDVTEEYMKSVADLMVLKGRPHFTVVRTFLVSDVTRGGFGEVDFGWGKAVYGGPAKGGVG-
PhAAT   321 EKLCKNPLGYALELVKKTKSDVTEEYMKSVADLMVIKGRPHFTVVRTYLVSDVTRAGFGEVDFGWGKAVYGGPAKGGVG-

McAAT   397 NIPHISSFHIRFRNAQGEERTMVSICLPHTAMERFVAEFESMMKEPIVSS-----TYKFTTSAL
CmAAT   396 AIPGVASFYIPFKNKKGERGILVPLCLPAPAMERFVKELDALLKAGKTIDGVDNKKPLFIASAL
NtAAT   400 AIPGVASFYIPFKNKKGENGIVVPICLPGFAMETFVKELDGMLKVDAPLD---NSNYAIIRPAL
PhAAT   400 AIPGVASFYIPFRNKKGENGIVVPICLPGFAMEKFVKELDSMLKGDAQLD---NKKYAFITPAL
```

Figure S3. (Continued). f. Multiple sequence alignment of deduced amino acid sequences of two representatives, branched-chain amino-acid aminotransferase (BCAT) and alcohol acyl transferase (AAT), from amino acid catabolic pathway.

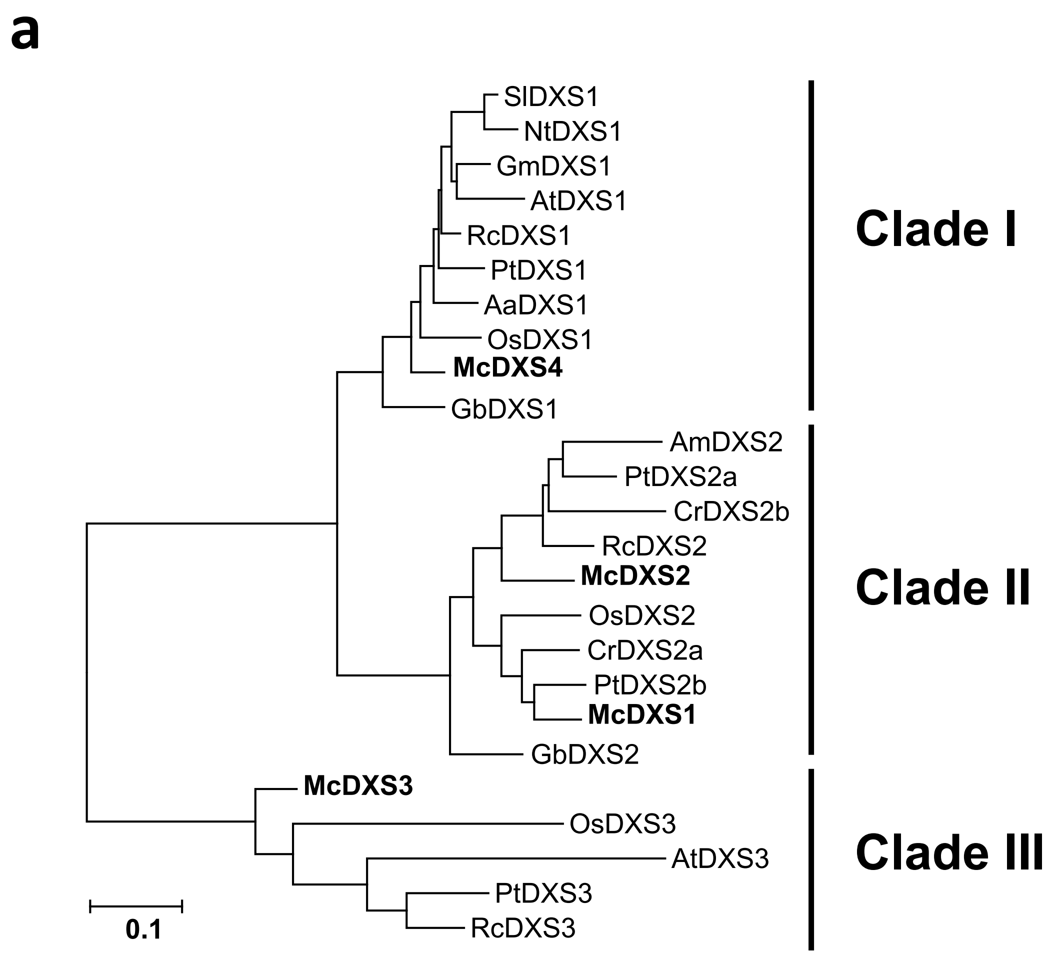

Figure S4. Phylogenetic analysis and amino acid alignment of champak DXSs. a. A maximum likelihood tree was constructed by MEGA 6 program from an alignment of McDXSs with other plant DXSs. Accession numbers of proteins used in McDXS phylogenetic analysis are given in Additional file 1: Table S6.

b

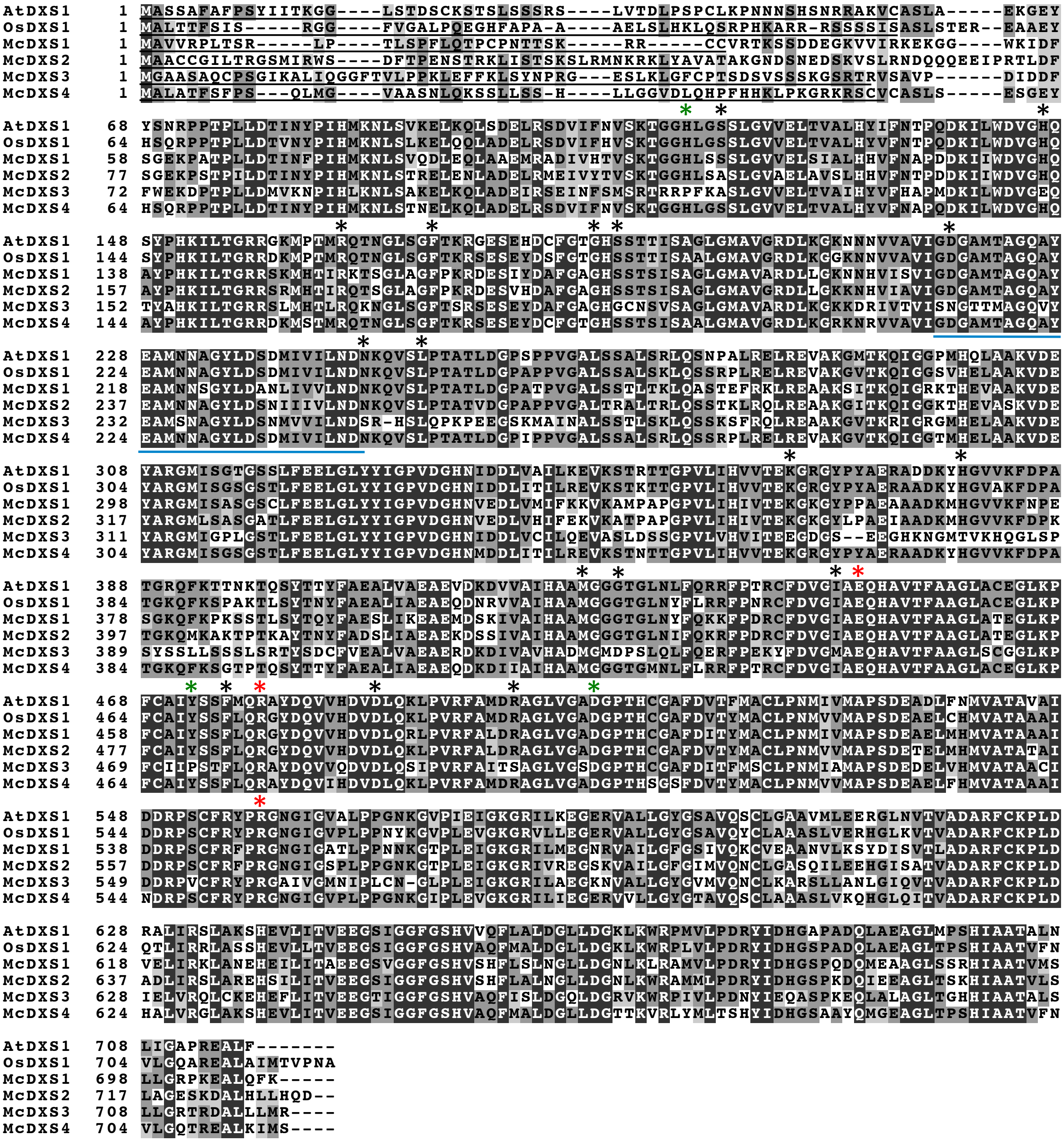

Figure S4. (Continued). b. Amino acid sequence alignment of McDXSs with DXS1 from *Arabidopsis thaliana* (AtDXS1; Q38854) and rice (OsDXS1; NP_001055525). Completely conserved amino acids are shown in white with black background. Residues conserved in four sequences are shown in black with grey background. The predicted sequences of plastidial transit peptides are underlined in black. The thiamine pyrophosphate (TPP) binding domain is underlined in blue. Asterisks mark the residues located in the active site (black), essential for glyceraldehyde-3-phosphate binding (green) and involved in enzyme catalysis (red).

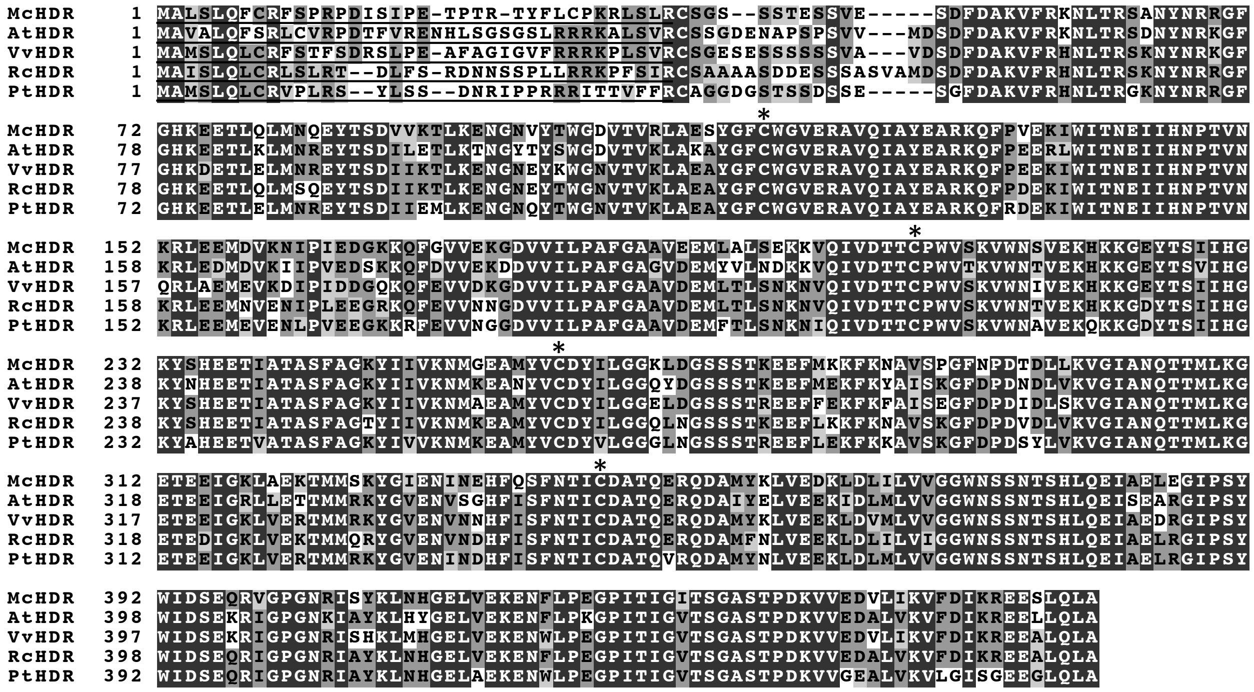

Figure S5. Amino acid sequence alignment of McHDR. Amino acid sequence of McHDR was aligned with HDRs from *Arabidopsis thaliana (*AtHDR; NP_567965), *Vitis vinifera* (VvHDR; XP_002284659), *Ricinus communis (*RcHDR; XP_002519102) and *Populus trichocarpa (*PrHDR; XP_002305413). Completely conserved amino acids are shown in white with black background. Residues conserved in four sequences are shown in black with grey background. The predicted sequences of plastidial transit peptides are underlined in black. Iron-sulfur cluster binding sites are marked by asterisk.

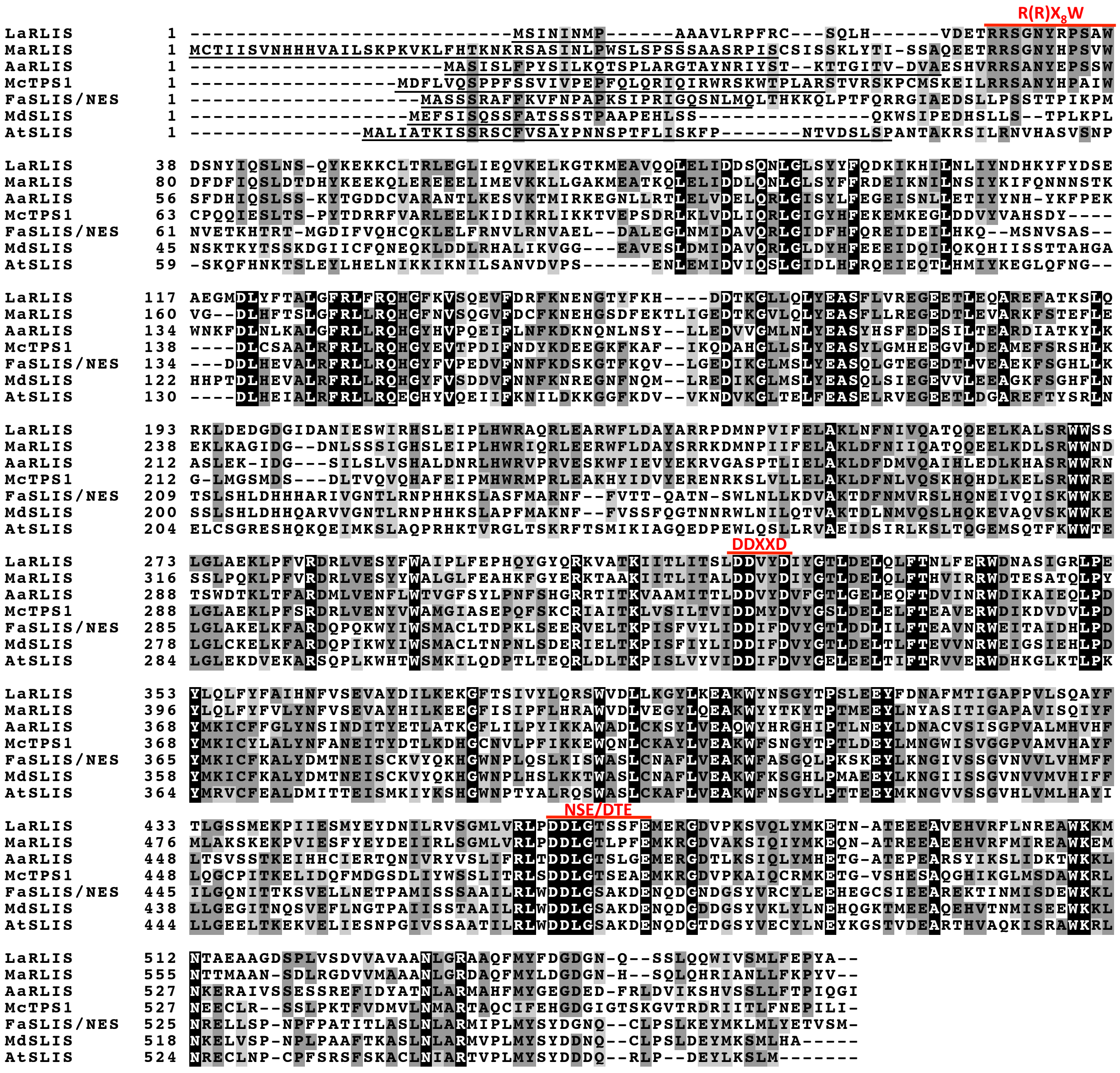

Figure S6. Amino acid sequence alignment of McTPS1. Amino acid sequence of McTPS1 was aligned with other linalool synthases from *Mentha aquatica* (MaRLIS; Q8H2B4), *Lavandula angustifolia* (LaRLIS; Q2XSC5), Artemesia annua (AaRLIS; Q9SPN1), *Malus domestica* (MdSLIS; NP_001280833), *Fragaria x ananassa (*FaSLIS/NES; P0CV95) and *Arabidopsis thaliana* (AtSLIS; NP_176361). The domains essential for TPS activity, R(R)X_8W motif, the Asp-Rich DDXXD motif and the NSE/DTE motifs are highlighted in red. Completely conserved amino acids are shown in white with black background. Residues conserved in four sequences are shown in black with grey background. The predicted sequences of plastidial transit peptides are underlined in black.

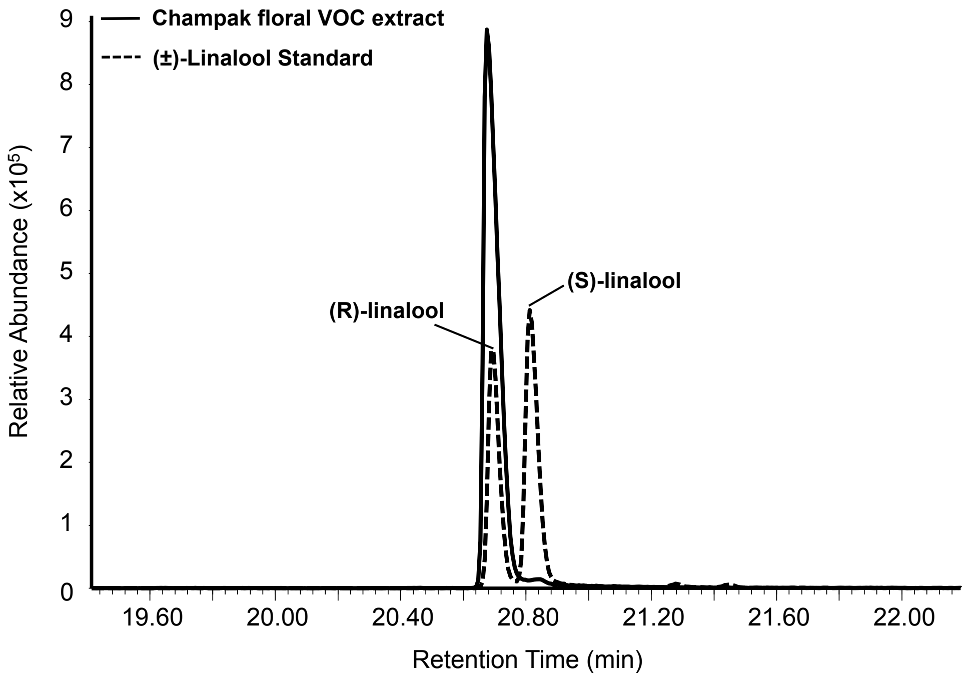

Figure S7. GC-MS chiral analysis of β-linalool emitted from champak flowers. VOCs were collected from headspace of champak flowers and analyzed by chiral gas chromatography. Authentic (R)-linalool and (S)-linalool were used for determination of enantiomeric distribution of linalool in champak flowers.

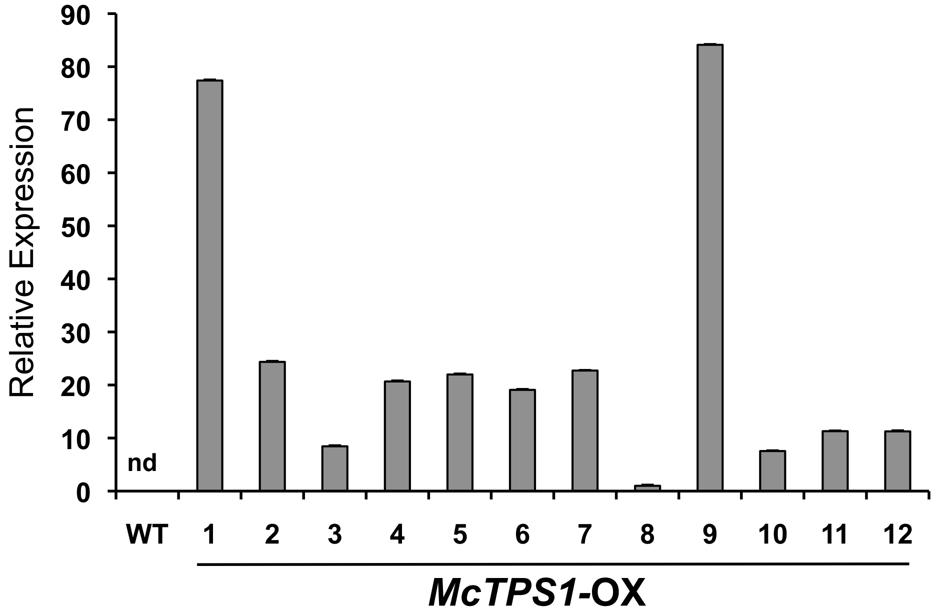

Figure S8. qRT-PCR analysis showing expression levels of *McTPS1* in independent lines of transgenic *N. tabacum* plants (lines #1-12, McTPS1-OX). WT, wild type; nd, not detectable.

Table S1. MEP pathway genes from champak RNA-seq

Gene	$\log_2$ (FPKM)	Transcript length (nt)	Deduced protein full length (aa)	Predicted transit peptide length (aa)	% Similarity to top BLASTX hit
McDXS1	10.19	7158	709	32	89
McDXS2	7.95	3328	731	45	85
McDXS3	7.93	1731	720	61	79
McDXS4	3.28	1396	677	53	91
McDXR	9.87	1979	471	47	93
McCMS	8.99	1622	321	68	76
McCMK	8.34	2502	409	59	82
McMCS	8.43	1126	236	56	88
McHDS	10.61	2841	743	64	92
McHDR	10.90	1994	460	37	87

Table S2. TPS genes from champak RNA-seq

No.	Length (nt)	$\log_2$(FPKM)	BLASTX top hit	% Similarity
1	1025	2.59	Delta-cadinene synthase (*Theobroma cacao*)	38
2	2030	2.26	Trans-ocimene synthase (*Litsea cubeba*)	67
3	1204	1.97	Beta-cubebene synthase (*Magnolia grandiflora*)	90
4	386	1.93	Beta-cubebene synthase (*Magnolia grandiflora*)	98
5	1215	1.42	Alpha-terpineol synthase (*Magnolia grandiflora*)	58
6	772	0.56	Beta-cubebene synthase (*Magnolia grandiflora*)	57
7	263	0.00	Beta-cubebene synthase (*Magnolia grandiflora*)	74
8	247	0.00	S-(+)-linalool synthase (*Cinnamomum osmophloeum*)	75
9	444	0.00	R-linalool synthase (*Morus notabilis*)	65

Table S3. Accession numbers of proteins used in the TPS phylogenetic analysis

Enzyme	Accession No.	Species	Function
**TPS-a**			
AaADS	Q9AR04	*Artemisia annua*	Amorpha-4,11-diene synthase
AaFS	AAX39387	*Artemisia annua*	(E)-beta-farnesene synthase
GaCad1-C1	AAD51718	*Gossypium arboreum*	(+)-delta-cadinene sythase
SlTPS9	NP_001234060	*Solanum lycopersicum*	Germacrene C synthase
VvCarS	AEP17005	*Vitis vinefera*	(E)-beta-caryophyllene synthase
**TPS-b**			
AaQH5	AAF13356	*Artemesia annua*	(3R)-linalool synthase
LaLINS	ABB73045	*Lavandula angustifolia*	(3R)-linalool synthase
LcTPS1	AEJ91554	*Litsea cubeba*	Trans-ocimene synthase
McLINS	AAL99381	*Mentha citrata*	(3R)-linalool synthase
MsLIMS	AAC37366	*Mentha spicata*	4S-limonene synthase
SoSCS	O81191	*Salvia officinalis*	1,8-cineole synthase
SoSSS	O81193	*Salvia officinalis*	(+)-sabinene synthase
VvMyrS	XP_002276045	*Vitis vinefera*	Myrcene synthase
**TPS-c**			
AtGA1	NP_192187	*Arabidopsis thaliana*	Copalyl pyrophosphate synthase
SlCPS	BAA84918	*Solanum lycopersicum*	Copalyl pyrophosphate synthase
**TPS-d**			
Ag2	O24474	*Abies grandis*	Myrcene synthase
Ag3	O24475	*Abies grandis*	(-)-(1S,5S)-pinene synthase
Ag9	Q9M7D0	*Abies grandis*	Terpinolene synthase
Ag10	O22340	*Abies grandis*	(-)-(4S)-limonene synthase
**TPS-e**			
SlSBS	XP004244438	*Solanum lycopersicum*	Santalene and bergamotene synthase
SrKS1-1	AAD34294	*Stevia rebaudiana*	Kaurene synthase
**TPS-f**			
CbLis	AAC49395	*Clarkia breweri*	(3S)-linalool synthase
**TPS-g**			
Am1e20	Q84NC9	*Antirrhinum majus*	Myrcene synthase
AtTPS14	NP_176361	*Arabidopsis thaliana*	(3S)-linalool synthase
FaSLIS/NES	CAD57106	*Fragaria ananassa*	(3S)-linalool/(E)-nerolidol synthase
MdLIS	AGB14629	*Malus domestica*	(3S)-linalool synthase
ObGES	Q6USK1	*Ocimum basilicum*	Geraniol synthase

Table S4. List of primers used in this study

Name	Forward	Reverse
**for qRT-PCR**		
McDXR	GTGCTTAGTGCAGCCAATGA	GATGCTGCAGACCAGACGTA
McCMS	AAGGTCGAGTTTGCGAGAAA	TTGAACCATGCGACAGAAAG
McCMK	GTGCCCAACTGGTGAAGTTT	ACTCCCCGACATGAAAACAG
McMCS	TTGGGCAGATATTCCCAGAC	CCCTGATGGACTCCTTGTGT
McHDS	CACGAATCATGGAAGCCTTT	CTCCCAAGTGCAAGGGATAA
McAACT	TTTCTTGGCACCCTTTCATC	CCCTGATGCGCAGACTTTAT
McHMGS	CATAAGTGCGTCGGGATTCT	GCGACCAATTTGTCTTGGAT
McHMGR1	GCCTTCCATTGAGGTTGGTA	GAGCAGACATGAGGGAGAGC
McMVK	CAGAGCATGGGAGTCAGTCA	ACCAATTTCAATGCCTTTGC
McPMK	TTCGCTTTTCTCCTGCAGTT	TTTGAGGATCGGATTTTTGC
McMPDC	TGCTTGTCTCGTTTTTGCAC	TCACATCCAGACGCATCATT
McDAHPS	AGGCTCCTTGTGGTCTCAAA	GAGCTCCAGAGACTGGGATG
McDHQS	CAAGCCTTCCTCCTCCTTCT	GCTCTTTTGCTGCTTCTGCT
McSDH	CTGTAGGATGCTGCGATGAA	TGCCTTTCCTTGCTCTCAAT
McSK	GGCACAACGAAAACCAAAGT	AGCTTTGGACAGCACCATCT
McCM	TTTACCTGAGCCCATTTTGC	AGCCGTCGATCCACAATTAC
McCS	AGGGAGTGGTCGACAATGAG	TTTATCAAAGGCCGGTGAAC
McPAL	GTGAGCCAGGTGGCTAAGAG	CAAAAGCCCCAATCTTTTGA
McC4H	ACGTGTGGTGGAAGATGTGA	GCCTCAAGATGGGAATGAAA
McC3H	CACTCCAAAGAGGCTTGAGG	CTTGATGCCTTCTGTGACGA
McCOMT	TTTGGAATGAATGCGTTTGA	AAGCGTGACTCCAATTCCAC
Mc4CL	GCTTGAGGGGCTTCTTCTTT	TTACCCTCCGCAATTTCTTG
McCoAOMT	ATCAGATATTCCGGCACCAG	CCGATCTCCATCGTGTTCTT
McCCR	GTGAACCCTGTGCTGGTTTT	GAATGTGAGCCTCAGCAACA
McCAD	TAACGTTGGAGTGGGTGTGA	AATAGTGGTGCCGCTTGTTC
Mc9-LOX	GACAGCGGTCCACACCTAAT	CCCACCAACTCCAGTGAGAT
Mc13-LOX	GCTAGTGGGCCAATCACAAT	GATCTGGATCTCCCAGGTCA
McHPL	TCGGCTCGACTACTTCTGGT	CGAGGACGTCTTTCTTTTCG
McAOS	AGATGCTGTTCGGTTTCCAG	TTCTCCACCGTTGGATTCTC
McAOC	CATCAGCTGCTTCAAGACCA	CGCCGTGATTCCTATACGTT
McOPR	GGATTCAAACCCACACAACC	TACGCTCTCTTCCACGACCT
McBCAT	ATTGGGAGGAGCTTGGATTT	CTCAGCTCCCTTCTGCATTC
McBCKDC_E1	GTCTGATCCGGAGATTTGGA	GCATAGTGGGCTTTCTCAGC
McBCKDC_E2	CAGCAGACTCTGGGGTTTTC	CCCTCTTGCACAAACCATTT
McBCKDC_E3	CTTTGACGGAAATCCCAAAA	AACCCCGACGACCTTAGTCT
McAAT	GGCAAGCCTGTGCTAGTTTC	TACCGGCAAACGGGTAGTAG
McDXS1	GCCAACCTGATCGTCGTATT	CCTTGCGTATTCATCCACCT
McDXS2	TGGCAGCATGTTGTGGTATT	AATCATTTCCCTTGGCTGTG
McDXS3	GCAGCCTCTCTCGGACATAC	TGACAAACCAGCAGCAAAAG
McDXS4	GAGCAGCATGCAGTCACATT	TTTGGTAGGCATGCCATGTA
McHDR	GAACATGGGAGAGGCAATGT	TCAGCCAATTTCCCAATCTC
McTPS1	GTGCCCAATCACCAAAGAGT	AAGCCCTTGATGTGTCCTTG
McActin	CACCCTCAAATACCCCATTG	GTTCGCTTTTGGGTTGAGAG

Table S4. (Continued)

**Name**	**Forward**	**Reverse**
**for full-length cDNA cloning**		
McDXS1	AAAAAGCAGGCTTCATGGCAGTTGTTAGGCCTCTAACAT	AGAAAGCTGGGTGTCACTTGAACTGAAGAGCTTCCTT
McDXS2	AAAAAGCAGGCTTCATGGCAGCATGTTGTGGTATTCTGA	AGAAAGCTGGGTGTTAATCTTGGTGGAGAAGATGGAGA
McDXS3	CACCATGGGTGCTGCTTCTGCTCA	TTAGCGCATTAAAAGAAGGGCATCCCG
McDXS4	CACCATGGCCCTTGCCACATTCTCCTTTCCTA	CTAGTAGTGAGATGTCAGCATATATAGTCGTACCTTTGTAGTG
AtDXS1	AAAAAGCAGGCTTCATGGCTTCTTCTGCATTTGCTTTTCCTT	AGAAAGCTGGGTGTCAAAACAGAGCTTCCCTTGGT
McHDR	AAAAAGCAGGCTTCATGGCGCTCTCTCTCCAATTCT	AGAAAGCTGGGTGCTATGCTAACTGCAAGGATTCTTC
AtHDR	AAAAAGCAGGCTTCATGGCTGTTGCGCTCCAATTCA	AGAAAGCTGGGTGTCAAGCCAGCTGCAATAACTCTT
McTPS1	CACCATGGATTTTCTTGTTCAATCCCCAC	TCAAATTAAAATTGGTTCGTTGAATAATGTGATGATCC

Table S5. Accession numbers of proteins used in the amino acid sequence alignments

Enzyme name	Accession No.	Species
**DXR**		
HbDXR	ABD92702	*Hevea brasiliensis*
AaDXR	AID55340	*Actinidia arguta*
NtDXR	ADD82536	*Narcissus tazetta var. chinensis*
**MCS**		
MsMCS	AFB70982	*Mitragyna speciosa*
NtMCS	AHM22925	*Nicotiana tabacum*
HbMCS	BAF98294	*Hevea brasiliensis*
**AACT**		
TcAACT	XP_007049378	*Theobroma cacao*
PtAACT	XP_002308755	*Populus trichocarpa*
HbAACT	AFJ74323	*Hevea brasiliensis*
**MVK**		
MnMVK	XP_010105842	*Morus notabilis*
HbMVK	AIO11226	*Hevea brasiliensis*
PnMVK	AFN02124	*Panax notoginseng*
**DHQS**		
MtDHQS	XP_003612212	*Medicago truncatula*
VvDHQS	NP_001268092	*Vitis vinifera*
CsDHQS	AJA40945	*Camellia sinensis*
**SK**		
TcSK	XP_007031961	*Theobroma cacao*
PtSK	XP_006373127	*Populus trichocarpa*
GaSK	KHG07566	*Gossypium arboreum*
**4CL**		
Pt4CL	XP_006373451	*Populus trichocarpa*
Fm4CL	AHL44983	*Fraxinus mandshurica*
It4CL	ADG46006	*Isatis tinctoria*
**CAD**		
GaCAD	KHG04573	*Gossypium arboreum*
MnCAD	XP_010096287	*Morus notabilis*
OtCAD	ADO16245	*Ocimum tenuiflorum*
**AOS**		
LjAOS	ABC17856	*Lonicera japonica*
InAOS	BAK52267	*Ipomoea nil*
CsAOS	AHY03308	*Camellia sinensis*
**AOC**		
PsAOC	BAE45342	*Pisum sativum*
VrAOC	XP_014520963	*Vigna radiata var. radiata*
GmAOC	NP_001304386	*Glycine max*
**BCAT**		
GaBCAT	KHG02043	*Gossypium arboreum*
RcBCAT	XP_002530599	*Ricinus communis*
CsBCAT	ALN97496	*Cucumis sativus*
**AAT**		
CmAAT	AAW51125	*Cucumis melo*
NtAAT	Q8GT20	*Nicotiana tabacum*
PhAAT	AAT68601	*Petunia x hybrida*

Table S6. Accession numbers of proteins used in the DXS phylogenetic analysis

Enzyme name	Accession No.	Species
**DXS Clade I**		
AaDXS1	AAD56390	*Artemisia annua*
AtDXS1	Q38854	*Arabidopsis thaliana*
GbDXS1	AAS89341	*Ginkgo biloba*
GmDXS1	ACO72582	*Glycine max*
NtDXS1	CBA12009	*Nicotiana tabacum*
OsDXS1	NP_001055525	*Oryza sativa Japonica Group*
PtDXS1	XP_002312717	*Populus trichocarpa*
RcDXS1	XP_002516843	*Ricinus communis*
SlDXS1	AAD38941	*Solanum lycopersicum*
**DXS Clade II**		
AmDXS2	AAW28999	*Antirrhinum majus*
CrDXS2a	CAA09804	*Catharanthus roseus*
CrDXS2b	ABI35993	*Catharanthus roseus*
GbDXS2	AAR95699	*Ginkgo biloba*
OsDXS2	NP 001059087	*Oryza sativa Japonica Group*
PtDXS2a	XP 002331678	*Populus trichocarpa*
PtDXS2b	XP 002303416	*Populus trichocarpa*
RcDXS2	XP 002533688	*Ricinus communis*
**DXS Clade III**		
AtDXS3	AED91670	*Arabidopsis thaliana*
OsDXS3	BAA83576	*Oryza sativa Japonica Group*
PtDXS3	XP_002308644	*Populus trichocarpa*
RcDXS3	XP_002514364	*Ricinus communis*
